# Clinical trial results in context: comparison of baseline characteristics and outcomes of 38,510 RECOVERY trial participants versus a reference population of 346,271 people hospitalised with COVID-19 in England

**DOI:** 10.1186/s13063-024-08273-9

**Published:** 2024-06-29

**Authors:** Guilherme Pessoa-Amorim, Raphael Goldacre, Charles Crichton, Will Stevens, Michelle Nunn, Andy King, Dave Murray, Richard Welsh, Heather Pinches, Andrew Rees, Eva J. A. Morris, Martin J. Landray, Richard Haynes, Peter Horby, Karl Wallendszus, Leon Peto, Mark Campbell, Charlie Harper, Marion Mafham

**Affiliations:** 1 Clinical Trial Service Unit, Oxford Population Health, University of Oxford, Richard Doll Building, Old Road Campus, Roosevelt Drive, Oxford, OX37LF UK; 2 Medical Research Council Population Health Research Unit, Oxford Population Health, University of Oxford, Oxford, UK; 3 Big Data Institute, Oxford Population Health, University of Oxford, Oxford, UK; 4 National Perinatal Epidemiology Unit, Oxford Population Health, University of Oxford, Oxford, UK; 5 NHS DigiTrials, Leeds, UK; 6 NIHR Oxford Biomedical Research Centre, Oxford University Hospitals NHS Foundation Trust, Oxford, UK; 7 Centre for Tropical Medicine and Global Health, Nuffield Department of Medicine, University of Oxford, Oxford, UK; 8 International Severe Acute Respiratory and emerging Infections Consortium (ISARIC), University of Oxford, Oxford, UK; 9 Pandemic Sciences Centre, University of Oxford, Oxford, UK; 10 Department of Infectious Diseases and Microbiology, Oxford University Hospitals NHS Foundation Trust, Oxford, UK

**Keywords:** COVID-19, Randomised trials, Evidence translation, RECOVERY

## Abstract

**Background:**

Randomised trials are essential to reliably assess medical interventions. Nevertheless, interpretation of such studies, particularly when considering absolute effects, is enhanced by understanding how the trial population may differ from the populations it aims to represent.

**Methods:**

We compared baseline characteristics and mortality of RECOVERY participants recruited in England (*n* = 38,510) with a reference population hospitalised with COVID-19 in England (*n* = 346,271) from March 2020 to November 2021. We used linked hospitalisation and mortality data for both cohorts to extract demographics, comorbidity/frailty scores, and crude and age- and sex-adjusted 28-day all-cause mortality.

**Results:**

Demographics of RECOVERY participants were broadly similar to the reference population, but RECOVERY participants were younger (mean age [standard deviation]: RECOVERY 62.6 [15.3] vs reference 65.7 [18.5] years) and less frequently female (37% vs 45%). Comorbidity and frailty scores were lower in RECOVERY, but differences were attenuated after age stratification. Age- and sex-adjusted 28-day mortality declined over time but was similar between cohorts across the study period (RECOVERY 23.7% [95% confidence interval: 23.3–24.1%]; vs reference 24.8% [24.6–25.0%]), except during the first pandemic wave in the UK (March–May 2020) when adjusted mortality was lower in RECOVERY.

**Conclusions:**

Adjusted 28-day mortality in RECOVERY was similar to a nationwide reference population of patients admitted with COVID-19 in England during the same period but varied substantially over time in both cohorts. Therefore, the absolute effect estimates from RECOVERY were broadly applicable to the target population at the time but should be interpreted in the light of current mortality estimates.

**Trial registration:**

ISRCTN50189673- Feb. 04, 2020, NCT04381936- May 11, 2020.

**Supplementary Information:**

The online version contains supplementary material available at 10.1186/s13063-024-08273-9.

## Background

Randomised controlled trials (RCTs) are essential to reliably evaluate safety and efficacy of health interventions [1, 2]. The use of randomisation (with allocation concealment) minimises the risk of bias, but, inevitability, due to eligibility criteria, trial participants are rarely representative of the populations whose treatment they aim to inform. Nonetheless, the proportional estimates of treatment effects from the trial are usually generalisable to the broader population, unless there are good grounds for believing there may be systematic differences in the effectiveness of the intervention or in the biology of the target disease outside of the trial setting (e.g. the advent of a new variant that renders a pathogen resistant to the particular drug that was studied) [3]. However, the estimates of *absolute* harm and benefit generated by such trials may not be directly generalisable, and assessment of the absolute rates of the relevant outcomes in the target population is useful to understand the likely absolute effects of the intervention in clinical practice [4, 5].

The Randomised Evaluation of COVID-19 Therapy (RECOVERY) trial is a randomised, controlled, open-label, pragmatic, platform trial of potential therapies for patients hospitalised with COVID-19 [6]. Eligibility criteria were broad and simple (i.e. hospitalisation for suspected or confirmed COVID-19), and trial procedures streamlined to be feasible in local practice. Data collection by trial staff, using dedicated case report forms (CRF), focused on the minimum information needed and was complemented with extensive linkage to several healthcare systems data sources in the UK. The trial took place in all acute UK National Health Service (NHS) hospitals, and in several other countries globally.

Here, we aimed to compare the baseline characteristics (demographics and comorbidities) and all-cause 28-day mortality (the trial primary outcome) for RECOVERY participants with a reference population hospitalised with COVID-19, within England.

## Methods

### RECOVERY cohort

The RECOVERY trial design has been described previously [6]. Briefly, RECOVERY recruited patients admitted to hospital with confirmed or suspected COVID-19 who were considered suitable for inclusion by their attending clinical team. Recruitment was not targeted to any particular subgroups or aimed at achieving a representative sample of the target population; the aim was to recruit a large number of participants rapidly. Randomisation was performed via a short online CRF in which essential baseline data are collected. Follow-up data were collected using a simple CRF upon death, hospital discharge, or at 28 days from randomisation (whichever occurs sooner). In the UK, these data were complemented with linkage to national healthcare systems data sources. The protocol, data analysis plan, baseline characteristics and outcome derivation documentation, and published results are openly available at www.recoverytrial.net, and the trial is registered with ISRCTN (50189673) and ClinicalTrials.gov (NCT04381936). Written informed consent was obtained from all the patients or from a legal representative if they were unable to provide consent. The RECOVERY trial has been approved by the UK Medicines and Healthcare products Regulatory Agency and the Cambridge East Research Ethics Committee (reference 20/EE/0101).

For this analysis, we included all RECOVERY participants recruited in England who had not withdrawn consent and had available healthcare systems data on hospital admissions (Hospital Episode Statistics [HES]) [7], with or without mortality data from official death records (Civil Registrations) [8]. We excluded children aged < 16 years due to difficulties in accessing linked healthcare systems data in this group in RECOVERY. HES data contained information on admissions to all NHS hospitals in England (using standardised coding practices since the 1990s), namely admission and discharge dates and relevant diagnostic and procedure codes. Diagnostic codes are recorded using the *International Classification of Diseases and Related Health Problems, Tenth Revision* (ICD-10) clinical terminology and can be assigned a position from 1 to 20; codes in position 1 usually indicate the primary cause of admission (or main cause of extension of hospital stay) [9]. Civil Registrations included information on date of death and underlying and contributing causes of death (also coded using ICD-10). HES and Civil Registrations were linked and supplied by NHS England [10].

### Reference population

To derive a reference population of people hospitalised with COVID-19 in England (thus potentially eligible for RECOVERY), we used an anonymised database covering the entirety of England which includes linked HES and Civil Registrations data continuously collected since 1999. These data were linked and supplied by NHS England, and are analysed at the University of Oxford [11]. More information can be found in the NHS England Data Uses Register at http://digital.nhs.uk/services/data-access-request-service-dars/data-uses-register (reference: DARS-NIC-315419-F3W7K). Approval for the use of the datasets was provided by the Central and South Bristol Research Ethics Committee (ref 04/Q2006/176).

The reference population was ascertained based on the presence of a COVID-19 ICD-10 code (U071—‘COVID-19, Virus identified’, or U072—‘COVID-19, Virus not identified’) [12]. This approach was informed by preliminary cross-validation work (Additional file 1: Annex III) using linked HES and SARS-CoV-2 testing data for RECOVERY participants, which showed 92% of RECOVERY participants recruited in England with a positive SARS-CoV-2 test (as captured in NHS England’s COVID-19 Second Generation Surveillance System—SGSS dataset) [13] had an admission in the HES data which included one of these codes in the primary diagnostic position. We therefore restricted our reference population to individuals with relevant ICD-10 codes in the primary position to avoid inclusion of people in whom COVID-19 was not the main reason for care. The RECOVERY cohort is largely contained within the reference population, but given the anonymised nature of the national datasets it was not possible to identify them.

### Analysis period

For each individual in RECOVERY and the reference population, we assigned an index date as the start of the earliest HES episode with U071/U072 in the first diagnostic position. For RECOVERY participants with index dates before 1 March 2020 (indicating long episodes before inclusion in the study; *n* = 22) or no COVID-19 codes in their HES records (*n* = 1465), we used randomisation date as the index date. We then restricted our analysis period to index dates between 1 March 2020 and 30 November 2021 inclusive. These analyses were not extended beyond this time-point as the launch of the high-dose dexamethasone comparison in the UK (only suitable to patients with oxygen or ventilation requirements) resulted in more selected patient populations being included in the trial [14].

### Baseline characteristic and outcomes

We used HES data in both cohorts to extract baseline clinical characteristics and demographics including age, sex, ethnicity, deprivation (quintile of Index of Multiple Deprivation 2019) [15], geographical location, Charlson Comorbidity Score [16, 17] and its components, and Hospital Frailty Risk Score [18]. Comorbidities were defined as the presence of a relevant ICD-10 code in any diagnostic position recorded within 5 years before the index date (i.e. excluding the index episode). Further methodological details, including the ICD-10 codes used, are provided in Additional file 1: Annex I. Geographical location data (including for deprivation assessments) were extracted from HES records and ascertained from full postcode in the RECOVERY HES data and lower-super output area of the postcode in the national HES data.

For outcomes, we calculated all-cause mortality within 28 days using linked HES and Civil Registrations data. Ascertainment of fact and date of death was based on these linked data sources (derivation methodology described elsewhere) [19]. We considered death records occurring in either healthcare systems data source. We ignored reports of deaths of RECOVERY participants recorded only on the CRF data as there were no CRF data for the reference population.

### Statistical analyses

This analysis is limited to RECOVERY participants in England with available HES data. To assess how this selection may have affected the cohort characteristics, we first compared the characteristics of those recruited in England with those recruited in other UK nations (using CRF data for all characteristics except ethnicity, and healthcare systems data in each nation for ethnicity). We then compared the characteristics of RECOVERY participants recruited in England who had available HES data with those who did not (using CRF data for all characteristics except ethnicity, for which we used healthcare systems data from primary care).

We compared baseline characteristics and 28-day mortality of the RECOVERY cohort with those of the reference population, in each case restricted to England only. Age was stratified into 4 groups: < 60, 60–69, 70–79, and ≥ 80. We presented continuous parameters as mean with standard deviation (SD) or median with interquartile range (IQR) as appropriate (with visual assessment of frequency distribution for normality) and frequency counts and percentage distribution for categorical parameters. We compared age, sex, and region of residence by calculating a representativeness ratio—defined as the proportion of people within RECOVERY in each category divided by the proportion of people within the reference population in the same category—and presented these along with 95% confidence intervals [95% CI] [20]. We also calculated a recruitment ratio defined as number of individuals included in RECOVERY divided by the number of individuals in the reference population. We then aggregated individuals in each cohort into three-month periods and conducted the same calculations as above for each time period separately.

The primary RECOVERY trial outcome of 28-day all-cause mortality was calculated starting from the index date in both cohorts, overall and over time (by three-month periods). We presented crude and age- and sex-adjusted mortality rates with 95% CI, with adjustment performed using direct standardisation methods [20] (i.e. applying RECOVERY mortality rates to the reference population age and sex composition using the age groups mentioned above). Further methodological details are provided in Additional file 1: Annex I.

We used Stata v17/MP to derive baseline characteristics and outcomes in HES and Civil Registrations data in both cohorts and R v4.2.1 for all subsequent data management, statistical analysis, and plotting (further details are provided in Additional file 1: Annex I).

## Results

### Baseline characteristics

**Fig. 1:**
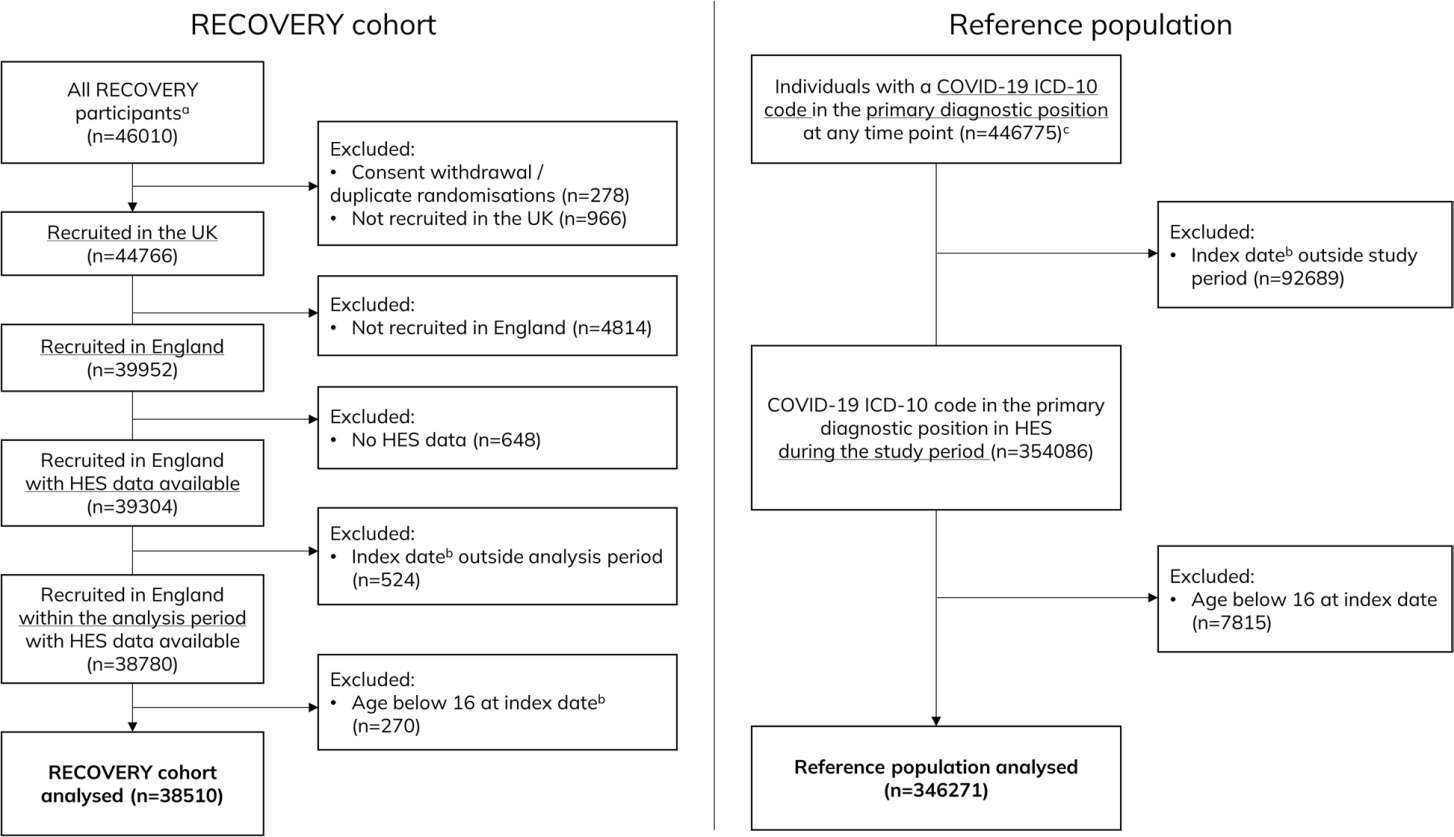
CONSORT diagram depicting the cohort derivation process. ‘^a^’ symbol indicates the following: randomised up until 1 September 2022. ‘^b^’ symbol indicates the following: index date is the episode start date for the earliest episode with a COVID-19 ICD-10 code in the primary diagnostic position. ‘^c^’ symbol indicates the following: up to June 2022 (latest data included in the raw extract)

**Fig. 2:**
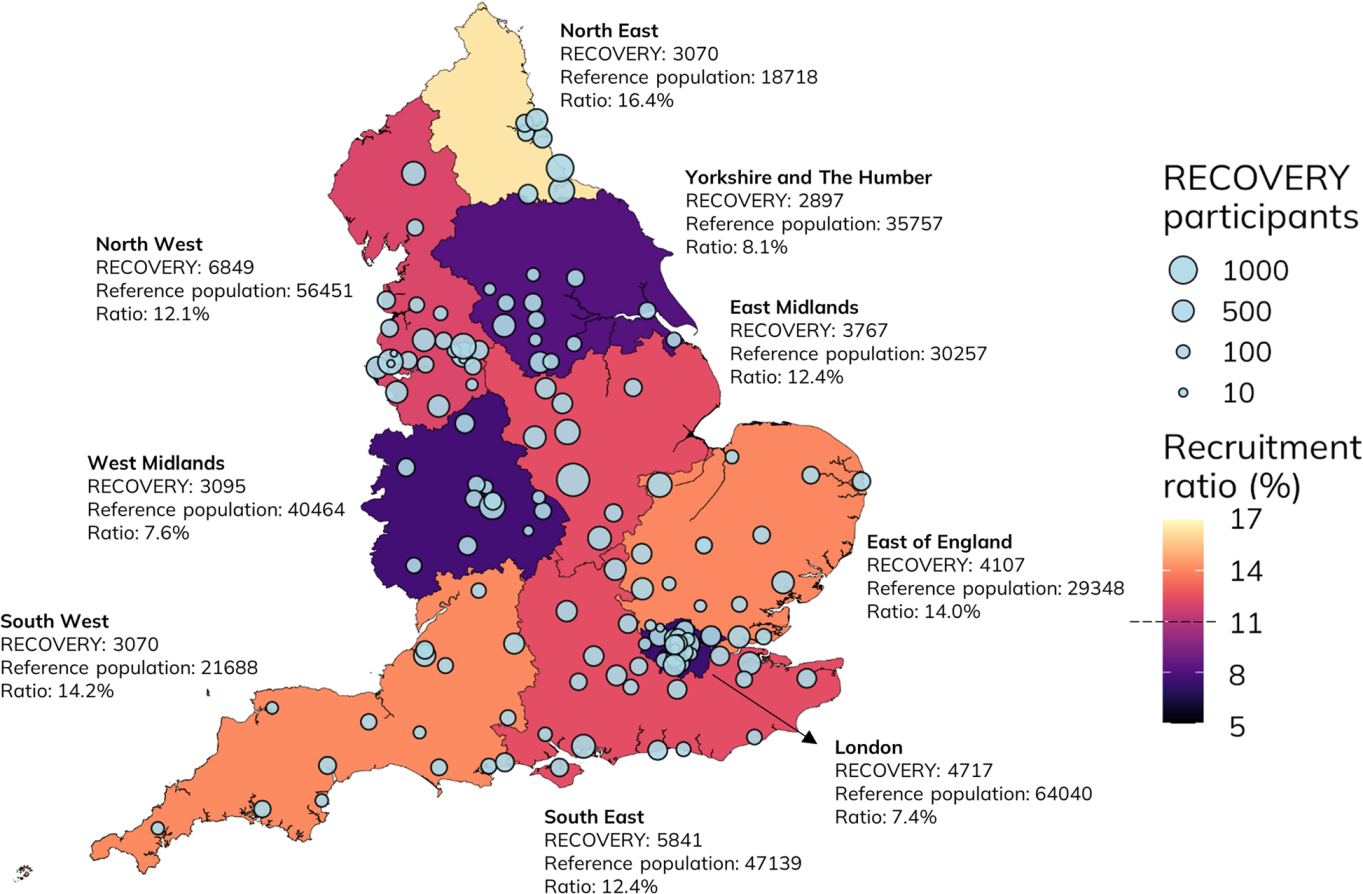
Geographical representativeness of the RECOVERY trial cohort in comparison with the national reference population. Number of RECOVERY participants plotted at the location of the recruiting NHS Trust hospital site. Recruitment ratios were calculated by dividing the number of RECOVERY participants recruited in each region by the number of individuals in the reference population in the same region and are presented by region. The average recruitment ratio across all English regions was 11.1%. There were 1097 and 2409 individuals with missing residential area in HES data in the RECOVERY and the reference population cohort, respectively

Up until 1st September 2022, RECOVERY recruited 46,010 participants, of which 44,766 in the UK and 39,952 in England. Of these, 39,304 (98.4%) had available HES data, and 38,780 were recruited within the analysis period (1 March 2020–30 November 2021). After excluding participants aged below 16 at the index date, a total of 38,510 participants were finally included in our analysis (Fig. 1). RECOVERY participants recruited in other UK nations had generally similar characteristics to those recruited in England (Additional file 1: Supplementary Table S1). People with no HES data available were younger, less frequently of white ethnicity, and had generally lower comorbidity burden and need for respiratory support at randomisation (Additional file 1: Supplementary Table S2). The reference population included 346,271 individuals (Fig. 1); for every 100 people admitted with COVID-19 in England, 11 participants were recruited to RECOVERY. When considering geographical region, the proportion of relevant patients recruited to RECOVERY in London, West Midlands, and Yorkshire and The Humber was lower than in the other England regions (Fig. 2).

Table 1 shows the baseline characteristics of both cohorts. RECOVERY participants were less frequently female (RECOVERY 37% vs reference population 45%) and were on average slightly younger than the reference population (mean age [SD]: 62.6 [15.3] vs 65.7 [18.5] years), with people aged 80 + and women underrepresented in RECOVERY throughout the analysis period (Additional file 1: Supplementary Figure S1 and Supplementary Table S3). RECOVERY participants were more frequently of White background (83% vs 79%) (Table 1 and Additional file 1: Supplementary Figure S2) but had similar deprivation status overall and throughout the study period (Additional file 1: Supplementary Figure S3).

**Table 1:** Baseline cohort characteristics

Characteristic	RECOVERY, *N* = 38,510	Reference population, *N* = 346,271
Age, mean (SD)	62.6 (15.3)	65.7 (18.5)
< 60	16,121 (41.9%)	123,790 (35.7%)
60–69	8906 (23.1%)	56,452 (16.3%)
70–79	7871 (20.4%)	69,107 (20.0%)
80 +	5612 (14.6%)	96,922 (28.0%)
Sex		
Female	14,060 (36.5%)	155,441 (44.9%)
Male	24,424 (63.5%)	190,748 (55.1%)
Geographical region^a^		
London	4717 (12.2%)	64,040 (18.5%)
North West	6849 (17.8%)	56,451 (16.3%)
South East	5841 (15.2%)	47,139 (13.6%)
West Midlands	3095 (8%)	40,464 (11.7%)
Yorkshire and The Humber	2897 (7.5%)	35,757 (10.3%)
East Midlands	3767 (9.8%)	30,257 (8.7%)
East of England	4107 (10.7%)	29,348 (8.5%)
South West	3070 (8%)	21,688 (6.3%)
North East	3070 (8%)	18,718 (5.4%)
Unknown/not resident in England	1097 (2.9%)	2409 (0.7%)
Ethnicity^a^		
White	29,595 (83.3%)	253,842 (78.9%)
Black	1171 (3.3%)	16,909 (5.3%)
Asian	3263 (9.2%)	35,785 (11.1%)
Other	1146 (3.2%)	11,853 (3.7%)
Mixed	351 (1.0%)	3532 (1.1%)
Unknown	2984 (7.7%)	24,350 (7.0%)
Index of multiple deprivation (quintile)		
1 (most deprived)	9821 (25.5%)	94,487 (27.3%)
2	8284 (21.5%)	78,400 (22.6%)
3	7466 (19.4%)	65,082 (18.8%)
4	6910 (17.9%)	57,203 (16.5%)
5 (least deprived)	5797 (15.1%)	48,650 (14.0%)
Unknown	232 (0.6%)	2449 (0.7%)
Charlson score, median (IQR)	3.0 (1.0, 5.0)	4.0 (1.0, 6.0)
Myocardial infarction	2941 (7.6%)	34,895 (10.1%)
Congestive heart failure	3158 (8.2%)	44,007 (12.7%)
Peripheral vascular disease	2052 (5.3%)	24,327 (7.0%)
Cerebrovascular disease	2603 (6.8%)	41,812 (12.1%)
Chronic pulmonary disease	8160 (21.2%)	80,492 (23.2%)
Rheumatic disease	1579 (4.1%)	17,365 (5.0%)
Dementia	1234 (3.2%)	30,314 (8.8%)
Peptic ulcer disease	710 (1.8%)	7693 (2.2%)
Liver disease (mild)	1515 (3.9%)	14,674 (4.2%)
Liver disease (moderate-severe)	175 (0.5%)	2490 (0.7%)
Diabetes mellitus (without chronic complications)	6056 (15.7%)	59,244 (17.1%)
Diabetes mellitus (with chronic complications)	1354 (3.5%)	16,111 (4.7%)
Chronic kidney disease	3800 (9.9%)	54,019 (15.6%)
Solid tumour	2463 (6.4%)	26,844 (7.8%)
Metastatic cancer	589 (1.5%)	8287 (2.4%)
Lymphoma	389 (1.0%)	3238 (0.9%)
Leukaemia	320 (0.8%)	2775 (0.8%)
AIDS/HIV^b^	0 (0.0%)	0 (0.0%)
Hospital frailty score, median (IQR)	5.1 (1.8, 11.4)	6.3 (1.8, 16.3)
High risk (> 15)	6737 (17.5%)	94,191 (27.2%)
Intermediate risk (5–15)	12,751 (33.1%)	99,402 (28.7%)
Low risk (< 5)	19,022 (49.4%)	152,678 (44.1%)
Other comorbidities/demographics		
Renal replacement therapy	439 (1.1%)	4922 (1.4%)
Immunosuppression	1471 (3.8%)	15,531 (4.5%)
Obesity	6147 (16.0%)	48,749 (14.1%)
Severe mental illness	4268 (11.1%)	43,969 (12.7%)
Alcohol-attributable diseases	945 (2.5%)	10,909 (3.2%)

Data are shown as mean (SD), *n* (%), or median (IQR)

*HES* Hospital Episode Statistics, *IQR* interquartile range, *SD* standard deviation

^a^Proportions for people with known and unknown geographical region and ethnicity were calculated separately, using the number with known region or ethnicity as the denominator for each category and the entire cohort as the denominator for those with unknown region or ethnicity

^b^ICD-10 codes for AIDS/HIV are censored from HES data

With respect to clinical conditions, RECOVERY participants had a lower prevalence of comorbidity (median Charlson Comorbidity Score [IQR]: RECOVERY 3.0 [1.0–5.0] vs reference population 4.0 [1.0–6.0]) and were less frail (median Hospital Frailty Risk Score [IQR]: 5.1 [1.8–11.4] vs 6.3 [1.8–16.3]) These differences were largely explained by the age structure of the two cohorts, with small differences remaining in the prevalence of some comorbidities, including cardiovascular disease, congestive heart failure, and dementia, after accounting for age (Additional file 1: Supplementary Figures S4-S6).

### Outcomes

**Fig. 3:**
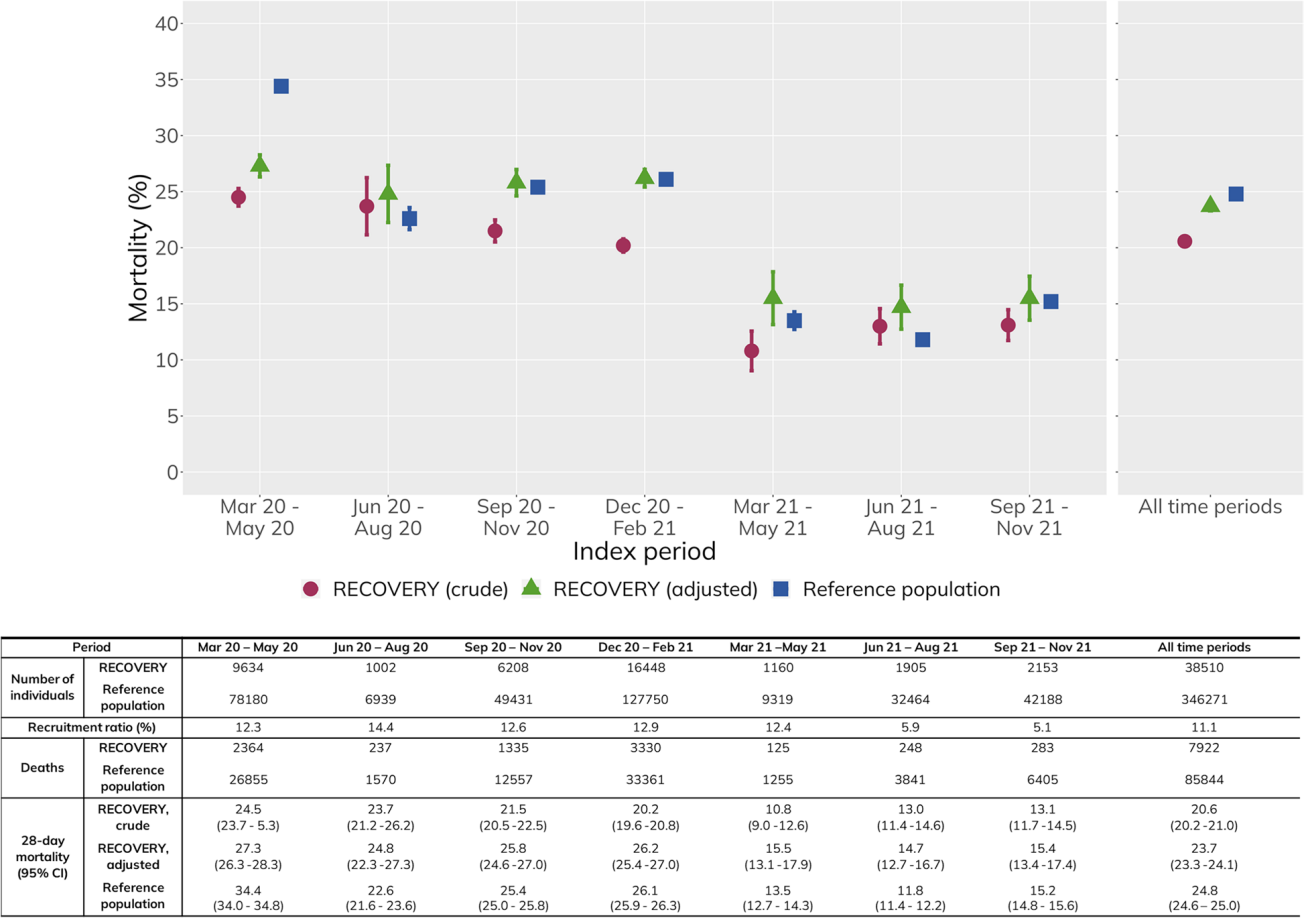
All-cause 28-day mortality over time in RECOVERY and the reference population. Twenty-eight-day mortality is the proportion of people with death recorded within 28 days of their index date (with 95% confidence intervals included). Adjustment performed by applying RECOVERY 28-day mortality to an age- (5-year bands) and sex-standardised population using the reference population, in a rolling basis within each time period (for 28-day mortality and age and sex breakdown)

Overall, the crude all-cause 28-day mortality in RECOVERY was 20.6% (95% CI: 20.2–21.0%) and 24.8% (95% CI: 24.6–25.0%) in the reference population, with mortality decreasing substantially in both cohorts from March 2021 onwards. After standardising the RECOVERY cohort to the age-sex composition of the national reference population, 28-day mortality in RECOVERY was similar to the reference population (23.7%, 95% CI: 23.3–24.1%; Fig. 3). Age-stratified mortality rates were similar between the two cohorts, with the exception of March–May 2020 where mortality was lower in RECOVERY (Additional file 1: Supplementary Figures S7-S8 and Supplementary Table S4). When mortality was assessed separately by comorbidity level and age, the difference in 28-day mortality between the two cohorts in March–May 2020 appeared to be mostly driven by older and more comorbid patients (Additional file 1: Supplementary Figure S9).

## Discussion

This study compared the characteristics of RECOVERY trial participants with people admitted to hospital due to COVID-19 in England. Our main findings were that RECOVERY participants were generally similar, but slightly younger, less frequently female, and had an overall lower comorbidity and frailty burden, much of which attributable to age differences. After adjustment for age and sex, 28-day mortality in the RECOVERY cohort was similar to that in the wider population of patients admitted to hospital with COVID-19 in England. This pattern was observed throughout the period studied, with the exception of March–May 2020 (corresponding to the first COVID-19 wave in the UK) when, even after adjusting for age and sex, 28-day mortality in RECOVERY was slightly lower than the reference population. The reasons for this are not fully explained by differences in measured frailty or comorbidity as assessed in our analyses and may be attributable to factors not captured in the datasets available in this study.

Older adults are frequently underrepresented in trials [21] and have been excluded from over half of COVID-19 clinical trials and all major vaccine trials [22]. Although RECOVERY does not have an upper age limit (and some participants were aged over 100 years old), in our study, RECOVERY participants were on average 3 years younger, with underrepresentation of people aged ≥ 80. RECOVERY participants were also less frequently female (37% vs 45%), but it is not possible to identify the underlying reasons for this in the available data. However, this is similar to results found in other trials and may be due to under-recruitment of older patients (who are more frequently women) [23, 24]. Of note, we found important differences in recruitment across different geographical regions, with the recruitment ratio (the number of individuals included in RECOVERY divided by the number of individuals in the reference population) ranging from 7.4 to 16.4%. The reasons for this are likely to be complex, including issues related to local research infrastructure and funding, competing studies, demand on local clinical services and clinician and patient willingness to engage with research. Data on these parameters are not available for this study, but these differences merit further investigation. We also found that comorbidity and frailty scores were lower in the RECOVERY cohort compared with the reference population. Most of these differences were attributable to age composition, but within older age groups, comorbidities and the overall frailty risk scores remained slightly higher in the reference population. Clinical decision making about eligibility for randomised trials will inevitably result in differences between the trial cohort and the target population; however, the proportional estimates of treatment effect from trials are usually generalisable, unless there are substantial differences in the biology of the target disease or the effectiveness of the intervention in the non-trial context [4, 5].

While crude 28-day all-cause mortality was lower in RECOVERY, age- and sex-adjusted mortality were generally similar, with similar trends in both cohorts over time. The reduction seen from March 2021 onwards, consistent with previous reports [25], may represent the effect of SARS-CoV-2 vaccination uptake, which greatly reduced the likelihood not only of hospital admission but also of death following hospitalisation [26, 27]. Overall, the absolute effect estimates generated by RECOVERY were generalisable to the national population during the period studied. However, secular trends in mortality rates should be considered and the best estimate of the likely absolute effect size in current clinical practice requires application of the proportional treatment effect from the RECOVERY trial to current absolute event rates among patients hospitalised with COVID-19 [4, 5].

Our study has a number of limitations. We were not able to determine baseline respiratory status (which has been shown to be an important determinant of the proportional and absolute benefits of corticosteroid treatment) [28] in our reference cohort, since there was low agreement between respiratory support status extracted from HES alone and that collected in the trial (based on a larger number of linked data sources) and used in published analyses (Additional file 1: Annex IV). We also cannot be certain whether our reference population had clinically significant COVID-19, although we have mitigated this by including only people with a relevant ICD-10 code in the primary diagnostic position. Our analysis was restricted to people admitted in England. Baseline characteristics were similar when comparing RECOVERY participants recruited across all UK nations, but may differ from non-UK countries. Finally, our analysis was restricted to the period from March 2020 to November 2021, due to changes to trial eligibility which could not be replicated in the reference population with the available data. However, recruitment to RECOVERY declined significantly from December 2021 onwards (along with national COVID-19 admissions), so that extending the analysis period to the time of writing would add only a small number of additional deaths (~ 4%), which were unlikely to meaningfully influence interpretation of our results.

## Conclusion

The RECOVERY trial recruited a broad patient population that was generally representative of people admitted to hospital due to COVID-19 in England during the same period, with respect to both baseline characteristics and subsequent mortality. Twenty-eight-day mortality declined substantially in both the RECOVERY and reference populations throughout the period studied. Estimates of current mortality rates from healthcare systems data combined with the proportional treatment effects from trials are needed to estimate the likely absolute effects of the treatments tested within current practice.

### Supplementary Information

Additional file 1: Annex I. Supplementary methods. Annex II. Supplementary tables and figures. Supplementary Figure S1. Age and sex representativeness of RECOVERY in comparison with the reference population. Supplementary Figure S2. Ethnicity over time in the RECOVERY cohort in comparison with the reference population, by age. Supplementary Figure S3. Deprivation over time in the RECOVERY cohort in comparison with the reference population, by age. Supplementary Figure S4. Charlson Comorbidity Score (excluding age) and Hospital Frailty Risk Score in the RECOVERY cohort and the reference population, by age groups. Supplementary Figure S5. Hospital Frailty Risk Score over time in the RECOVERY cohort and the reference population, by age groups. Supplementary Figure S6. Prevalence of select comorbidities in the RECOVERY cohort and reference population, by age groups. Supplementary Figure S7. 28-day mortality in the RECOVERY HES and All-England HES populations (stratified by age and sex). Supplementary Figure S8. 28-day mortality over time in RECOVERY and the reference population (by age groups). Supplementary Figure S9. Mortality rates over time in RECOVERY and the reference population, by age, comorbidity, and frailty. Supplementary Table S1. Baseline characteristics of the RECOVERY population recruited in the UK, grouped by nation (using data from the case report form only, except where stated). Supplementary Table S2. Baseline characteristics of the RECOVERY population recruited in England, grouped by HES linkage status (using data from the case report form only, except where stated). Supplementary Table S3. Number of individuals included in RECOVERY versus reference population over time, by age groups. Supplementary Table S4. 28-day mortality along time in the RECOVERY HES and All-England HES cohorts (split by age groups). Supplementary Table S5. Cross-coding of COVID-19 in HES and SGSS data within the RECOVERY population. Supplementary Table S6. Cross-coding of COVID-19 in HES (disaggregated across different ICD-10 codes) and SGSS data within the RECOVERY population. Supplementary Table S7. IMV coding cross tabulation (HES vs CRF)—15 days before randomisation. Supplementary Table S8. IMV coding cross tabulation (HES vs CRF)—30 days before randomisation. Supplementary Table S9. NIV coding cross tabulation (HES vs CRF)—15 days before randomisation. Supplementary Table S10. NIV coding cross tabulation (HES vs CRF)—30 days before randomisation. Annex III. Cross-coding of COVID-19 coding in Hospital Episode Statistics versus testing data in SGSS within the RECOVERY population. Annex IV. Cross-coding of invasive and non-invasive mechanical ventilation in the RECOVERY case report form versus Hospital Episode Statistics data

Additional file 2.

Additional file 3.

## Data Availability

The RECOVERY trial protocol, consent form, statistical analysis plan, definition and derivation of clinical characteristics and outcomes, training materials, regulatory documents, and other relevant study materials are available online at www.recoverytrial.net. Data will be made available in line with the Nuffield Department of Population Health policy and procedures. Those wishing to request access should complete the form available at http://www.ndph.ox.ac.uk/data-access and email it to data.access@ndph.ox.ac.uk. Nationwide anonymised English mortality (Civil Registrations) and hospitalisations (HES) data used to derive the reference population can be obtained upon application to NHS England at www.digital.nhs.uk. The statistical programming code used in this work is available for inspection and reuse at https://github.com/gpessoaamorim/recovery-generalizability-representativeness.

## Abbreviations

95% CI: 95% Confidence intervals
CRF: Case report form
HES: Hospital Episode Statistics
ICD-10: International Classification of Diseases and Related Health Problems, Tenth Revision
IQR: Interquartile range
NHS: National Health Service
RCTs: Randomised controlled trials
RECOVERY: Randomised Evaluation of COVID-19 Therapy
SD: Standard deviation
