# Supplementary material for: Clinical trial results in context: comparison of baseline characteristics and outcomes of 38,510 RECOVERY trial participants versus a reference population of 346,271 people hospitalised with COVID-19 in England
Source: Trials. 2024 Jun 29;25:429. doi: 10.1186/s13063-024-08273-9 (PMC11218071; doi:10.1186/s13063-024-08273-9)
Supplement: Supplementary file 1 — Additional file 1: Annex I. Supplementary methods. Annex II. Supplementary tables and figures. Supplementary Figure S1. Age and sex representativeness of RECOVERY in comparison with the reference population. Supplementary Figure S2. Ethnicity over time in the RECOVERY cohort in comparison with the reference population, by age. Supplementary Figure S3. Deprivation over time in the RECOVERY cohort in comparison with the reference population, by age. Supplementary Figure S4. Charlson Comorbidity Score (excluding age) and Hospital Frailty Risk Score in the RECOVERY cohort and the reference population, by age groups. Supplementary Figure S5. Hospital Frailty Risk Score over time in the RECOVERY cohort and the reference population, by age groups. Supplementary Figure S6. Prevalence of select comorbidities in the RECOVERY cohort and reference population, by age groups. Supplementary Figure S7. 28-day mortality in the RECOVERY HES and All-England HES populations (stratified by age and sex). Supplementary Figure S8. 28-day mortality over time in RECOVERY and the reference population (by age groups). Supplementary Figure S9. Mortality rates over time in RECOVERY and the reference population, by age, comorbidity, and frailty. Supplementary Table S1. Baseline characteristics of the RECOVERY population recruited in the UK, grouped by nation (using data from the case report form only, except where stated). Supplementary Table S2. Baseline characteristics of the RECOVERY population recruited in England, grouped by HES linkage status (using data from the case report form only, except where stated). Supplementary Table S3. Number of individuals included in RECOVERY versus reference population over time, by age groups. Supplementary Table S4. 28-day mortality along time in the RECOVERY HES and All-England HES cohorts (split by age groups). Supplementary Table S5. Cross-coding of COVID-19 in HES and SGSS data within the RECOVERY population. Supplementary Table S6. Cross-coding of COVI [file 13063_2024_8273_MOESM1_ESM.docx]

# Supplementary data

## Annex I – Supplementary methods

**ANALYSIS POPULATIONS AND TIMEFRAMES**

1. RECOVERY cohort

- All RECOVERY participants recruited in England for whom linked HES data is available (regardless of the presence of COVID codes)

1. Reference population

- All individuals in England with a relevant COVID-19 ICD-10 code in the primary diagnostic position (U071 or U072), in any episode during the study period

Data linkage for RECOVERY was performed by NHS Digital using a unique patient identifier (NHS number) along with name, gender, and date-of-birth where necessary. Data linkage (between sources) for the reference population was also performed by NHS Digital, and data were provided in an anonymised fashion.

We did not take COVID-19 ICD-10 coding characteristics into consideration when defining the RECOVERY cohort (i.e. matching these to the rules used to derive the reference population) as the purpose of this analysis was not to create perfectly matched populations, but rather highlight any potential differences between the RECOVERY cohort and a reference population.

An index date was defined for each individual as the *epistart* (in HES) for the first episode with an ICD-10 COVID-19 code (U071/U072) in the first diagnostic position in HES.

The analysis populations were restricted to individuals with an index date before the 1^st^ December 2021 (so that a 28 day follow-up period will have been completed by the end of December 2021). This allowed at least a two-month interval between reception of linked datasets in the first quarter of 2022 and the end of the follow-up period. Mortality was assessed within 28 days after the index date, with randomisation counted as day 1 and including day 28 (i.e. last index date is 30/11/21 and last day for outcome assessments is 27/12/21, inclusive).

We used the presence of a single COVID-19 ICD-10 code to define the reference population, following preliminary work exploring cross-coding of ICD-10 codes (in HES) and SARS-CoV-2 testing (from the Second Generation Surveillance System – SGSS data) in the RECOVERY population - which may be interpreted as a population with a proven or suspected clinical diagnosis of COVID-19. This work showed high agreement between COVID-19 coding in HES and a positive COVID-19 test in SGSS (see Annex IV)

No sampling or matching method was used to define the reference population as the purpose of this study is to compare how representative the RECOVERY population is in comparison with the national population (sampling would reduce these differences). However, a quantitative assessment of the generalisability of the trial results was performed by presenting age- and sex-adjusted all-cause 28 day mortality (i.e. the RECOVERY trial primary outcome), overall and over time.

Due to ethical considerations surrounding the ongoing collection of linkage data on people below the age of 16 in RECOVERY, we excluded individuals aged below 16 at the index date from all cohorts.

**DATA SOURCES AND DATA CUTS**

1. RECOVERY:
   1. Hospital Episode Statistics + Civil Registrations data (RECOVERY extract 79, received September 2022)
2. Reference population:
   1. Hospital Episode Statistics + Civil Registrations data - these data are part of an existing anonymised data flow provided to the University of Oxford by NHS Digital for public health research and held by the Unit of Healthcare Epidemiology at the University of Oxford (reference: DARS-NIC-315419-F3W7K); see the NHS Digital data uses register at <https://digital.nhs.uk/services/data-access-request-service-dars/data-uses-register?msclkid=480bee5ab0e111ec99ed4c48b4e33bc8> for more details on the data used

**BASELINE CHARACTERISTICS**

1. **Clinical conditions and general demographics**

Clinical conditions and demographics were derived from HES data. When comparing RECOVERY participants recruited in England vs other nations, and those with available HES data vs those without, we also used CRF recorded date. These conditions were defined based on recorded answers in the baseline CRF data, or the presence/absence of a relevant clinical code recorded within 5 years before the index date in HES. Respiratory status at baseline for RECOVERY trial participants was derived using a combination of data sources as described in the RECOVERY trial website (<http://www.recoverytrial.net/results>)

The exceptions to these rules are the following:

1. **Immunosuppression**, defined based on the presence of any of the COVID-19 Greenbook Immunisation Criteria^1^ as specified below:

• Cancer undergoing active chemotherapy: Cancer diagnosis ICD-10 code in any diagnostic position in the 5 years prior to randomisation AND chemotherapy ICD-10 or OPCS-4 code in any diagnostic position in the 6 months prior to randomisation.

• Haematological malignancy or bone marrow transplant: Haematological malignancy or transplant ICD-10 or OPCS-4 code in any diagnostic position in the 2 years prior to randomisation

• Solid organ transplant: solid organ transplant ICD-10 or OPCS-4 code in any diagnostic position in the 5 years prior to randomisation

• Hyposplenism: hyposplenism ICD-10 or splenectomy OPCS-4 code in any diagnostic position in the 5 years prior to randomisation

• Other immunosuppression: Other long-term immunosuppression ICD-10 in any diagnostic position in the 5 years prior to randomisation or other short-term immunosuppression ICD-10 or OPCS-4 code in any diagnostic position in the 6 months prior to randomisation

1. **Renal replacement therapy,** defined based on the presence of any of the criteria below in HES data only (using an adaptation of a previously-published algorithm):^2^

- Peritoneal dialysis: occurrence of any admission with a peritoneal dialysis code (without diagnosis of acute kidney injury) in the 5 years before index date (epistart of the index spell)
- Maintenance haemodialysis: Occurrence of a dialysis code in the 5 years before index date (*epistart* of the index spell) of the index spell in a patient who has had:

(a) a diagnostic code for ESKD any prior time, or within 365 days; OR

(b) the insertion of an arteriovenous fistula or graft any prior time, or within 365 days.

- Probable maintenance haemodialysis: Occurrence of at least two episodes containing a dialysis code, with at least 90 days between the start of the first recorded dialysis, and the start of any subsequent dialysis (without diagnosis of acute kidney injury) in the 5 years before index date (*epistart* of the index spell)

1. **Charlson Comorbidity Score (CCS):** scoring was derived using previously-published methodology^3^
2. **Hospital Frailty Risk Score:** the score wase assessed using HES data only, and only records with *epistart* in the 2 years preceding index date (following the published methodology);^4^ scoring of each item will be based on the same paper. NB: although the score was developed and validated in a population of over 75 years, it will be assessed here in the entire HES cohorts regardless of age
3. **Ethnicity:** ethnicity was derived in line with methodology used in the main RECOVERY publications (<http://www.recoverytrial.net/results>) and categorised according to the UK Department of Health categories (http://www.ethnicity-facts-figures.service.gov.uk/style-guide/ethnic-groups); in short, we used HES data only and selected the most frequent code in the ethnicity field. For comparisons of RECOVERY participants recruited in England versus other UK nations, we used all available data sources (which include HES and primary care data from the General Practice Extraction Service Data for Pandemic Planning and Research [GDPPR] in England, and datasets similar to HES in Scotland and Wales; no linkage data was available for Northern Ireland). In GDPPR, we selected the most frequent ethnicity SNOMED code in the journals table was used where available; if not, the most frequent record in the *ethnic* field in the patients table was used; for HES, the most frequent code in the ethnicity field was used as described above. All records were considered regardless of when they were recorded. If there was a tie, ethnic groups were prioritised in the following order (based on ascending recording frequency in the overall population): Mixed, Other, Black, Asian, White. Where there was a discrepancy between the best estimate from GDPPR and HES, the GDPPR group was prioritised.
4. **Codelist derivation**

Where possible, we used publicly-available clinical codelists to derive baseline characteristics using ICD-10 codes. For the remaining characteristics, original codelists were generated by one clinician by manually searching the ICD-10 terminology and reviewed by a second clinician. ICD-10 codelists to derive CCS,^3^ HFS,^4^ renal-replacement therapy,^2^ alcohol-attributable diseases,^5^ diabetes,^6^ severe mental illness,^7^ and obesity^8^ were taken from previously-published reports. Chronic heart disease was extracted with ICD-10 codes used to assign cardiac cause of death in the RECOVERY trial. Chronic liver disease was defined by merging codelists for mild and moderate-to-severe liver disease components of the Charlson score. The table below provides details on each HES-derived condition. All codelists used in this project are available for reuse and inspection at http://gitlab.ndph.ox.ac.uk/guilhermep/recovery-generalizability-representativeness/Tools.

**Hospital Episode Statistics derivation methodology and codelists**

Note – the HES APC data dictionary is available at <http://digital.nhs.uk/data-and-information/data-tools-and-services/data-services/hospital-episode-statistics/hospital-episode-statistics-data-dictionary#download-hes-data-dictionaries>

| **Characteristic** | **HES field** | **ICD-10 / OPCS-4 codes**  (NB: all codes are ICD-10 unless otherwise specified) | **Derivation rule** | **Notes** | **References** |
| --- | --- | --- | --- | --- | --- |
| Age | admiage | N/A | Value recorded in index spell |  | HES APC data dictionary |
| Gender | sex | N/A | Value recorded in index spell |  | HES APC data dictionary |
| Ethnicity | ethnos | N/A | Value recorded in index spell |  | HES APC data dictionary |
| COVID-19 coding | diag_01 to diag_20 (any position) | U071, U072 | If present in index spell (NB: the presence of these codes is used to define the national HES cohort; for the RECOVERY HES cohort fwe have used presence of a COVID-19 code, or else date of randomisation) | WHO documentation | <https://www.who.int/standards/classifications/classification-of-diseases/emergency-use-icd-codes-for-covid-19-disease-outbreak> |
| Index of multiple deprivation (score) | lsoa11 | N/A | Value recorded in index spell | Derived from the lsoa11 (lower-super-output-area) field rather than IMD as IMD provided in HES at the moment refers to the 2010 indices (most recent are 2019) | https://www.gov.uk/government/statistics/english-indices-of-deprivation-2019 |
| Index of multiple deprivation (quintiles) | lsoa11 | N/A | Value recorded in index spell |  |  |
| Charlson score (aggregated) | diag_01 to diag_20  (any position) | (see individual parameters) | Any record in the 5 years before index date (epistart of the index spell) | Charlson score calculated from ICD-10 codes using published and validated methodology and codelists | 10.1097/01.mlr.0000182534.19832.83 |
| Myocardial infarction (Charlson score) |  | I21, I22, I25.2 |  |  |  |
| Congestive heart failure (Charlson score) |  | I09.0, I11.0, I13.0, I13.2, I25.5, I42.0, I42.5, I42.6, I42.7, I42.8,  I42.9, I43, I50, P29.0 |  |  |  |
| Peripheral vascular disease (Charlson score) |  | I70, I71, I73.1, I73.8, I73.9, I77.1, I79.0, I79.2, K55.1, K55.8,  K55.9, Z95.8, Z95.9 |  |  |  |
| Cerebrovascular disease (Charlson score) |  | G45 |  |  |  |
| Chronic pulmonary disease (Charlson score) |  | I27.8, I27.9, J40, J41, J42, J43,  J44, J45, J46, J47, J60, J61, J62,  J63, J64, J65, J66, J67, J68.4, J70.1, J70.3 |  |  |  |
| Rheumatic disease (Charlson score) |  | M05, M06, M31.5, M32, M33, M34, M35.1, M35.3, M36.0 |  |  |  |
| Dementia (Charlson score) |  | F00, F01, F02, F03, F05.1, G30,  G31.1 |  |  |  |
| Peptic ulcer disease (Charlson score) |  | K25, K26, K27, K28 |  |  |  |
| Liver disease, mild (Charlson score) |  | B18, K70.0, K70.1, K70.2, K70.3, K70.9, K71.3, K71.4, K71.5, K71.7, K73, K74, K76.0, K76.2,  K76.3, K76.4, K76.8, K76.9,  Z94.4 |  |  |  |
| Liver disease, moderate-severe (Charlson score) |  | I85.0, I85.9, I86.4, I98.2,  K70.4, K71.1, K72.9, K76.5,  K76.6, K76.7 |  |  |  |
| Diabetes mellitus, with chronic complications (Charlson score) |  | E10.2, E10.3, E10.4, E10.5, E10.7, E11.2, E11.3, E11.4, E11.5, E11.7, E12.2, E12.3,  E12.4, E12.5, E12.7, E13.2,  E13.3, E13.4, E13.5, E13.7,  E14.2, E14.3, E14.4, E14.5,  E14.7 |  |  |  |
| Diabetes mellitus, without chronic complications  (Charlson score) |  | E10.0, E10.1, E10.6, E10.8,  E10.9, E11.0,E11.1, E11.6,  E11.8, E11.9, E12.0, E12.1,  E12.6, E12.8, E12.9, E13.0,  E13.1, E13.6, E13.8, E13.9,  E14.0, E14.1, E14.6, E14.8,  E14.9 |  |  |  |
| Renal disease  (Charlson score) |  | I21.0, I13.1, N03.2, N03.3, N03.4, N03.5, N03.6, N03.7,  N05.2, N05.3, N05.4, N05.5,  N05.6, N05.7, N18, N19, N25.0, Z49.0, Z49.1, Z49.2, Z94.0, Z99.2 |  |  |  |
| Any solid tumour, excluding malignant neoplasm of skin  (Charlson score) |  | C00-C26, C30-C34, C37-C41,  C43, C45-C58, C60—C76, C88.2, C88.7, C88.9, C90.0, C90.2, C90.3, C96-C97 |  |  |  |
| Metastatic solid tumour (Charlson score) |  | C77-C80 |  |  |  |
| Hemiplegia or paraplegia (Charlson score) |  | G04.1, G11.4, G80.1, G80.2, G81, G82, G83.0, G83.1, G83.2, G83.3, G83.4, G83.9 |  |  |  |
| AIDS /HIV  (Charlson score) |  | B20, B21, B22, B24 |  |  |  |
| Lymphoma  (Charlson score) |  | B21.1, C81-C86, C88.0, C88.3, C88.4 |  | Codelists built based on clinical review of the ICD-10 terminology by two clinicians (no publicly available codelists for these criteria) | Bespoke |
| Leukemia  (Charlson score) |  | C90.1, C91-C95, M36.1, D47.1, N16.1, D47.5 |  |  |  |
| Heart disease (any) | diag_01 to diag_20  (any position) | I08, I09, I11, I13, I20, I21, I22, I23, I24, I25, I27.1, I27.8, I27.9,  I30, I31, I32, I33, I34, I35, I36, I37, I38, I39, I40, I41, I42, I43, I44, I45, I46, I47, I48, I49, I50, I51, I52 | Any record in the 5 years before index date (epistart of the index spell) | RECOVERY trial cause-of-death derivation (“cardiac” category) | https://www.recoverytrial.net/results |
| Diabetes (any) |  | E10-E14 |  | Merged both Charlson diabetes codelists (codes above), manual review of the ICD-10 terminology, and literature review | https://bmjopen.bmj.com/content/6/8/e009952.long |
| Chronic liver disease |  | B18, K70.0, K70.1, K70.2, K70.3, K70.9, K71.3, K71.4, K71.5, K71.7, K73, K74, K76.0, K76.2,  K76.3, K76.4, K76.8, K76.9,  Z94.4, I85.0, I85.9, I86.4, I98.2,  K70.4, K71.1, K72.9, K76.5,  K76.6, K76.7 |  | Merged mild and moderate-severe liver disease lists | N/A |
| Severe mental illness |  | F20, F21, F22, F23, F24, F25, F28, F29, F30, F31, F32.2, F32.3  F32.8, F32.9, F33.2, F33.3  F33.4, F33.8, F33.9 |  | Publicly-available codelist used for the QCOVID NHS risk calculator | https://www.datadictionary.nhs.uk/Covid19PRA/Severe_Mental_Illness.html |
| Alcohol-attributable diseases |  | F10.3-F10.9, F10.0, F10.1,  F10.2, G62.1, G31.2, G72.1,  I42.6, K29.2, K70.0-K70.4,  K70.9, K85.2, K86.0, Q86.0,  P04.3 |  | Publicly-available codelist published by the US CDC | https://www.cdc.gov/alcohol/ardi/alcohol-related-icd-codes.html |
| Obesity |  | E66 | Any record in the 5 years before index date (epistart of the index spell) | ICD-10 terminology review, literature review | https://www.ncbi.nlm.nih.gov/pmc/articles/PMC6748036/ |
| Immunosuppression (cancer with active chemotherapy) | diag_01 to diag_20 for ICD-10 codes  (any position)  opertn_01 to opertn _21 for OPCS-4 codes (any position) | **Cancer (ICD-10):**  Check full list in codelist repository file (spreadsheet)  **Chemotherapy (ICD-10):**  Y43.1, Y43.3, Z51.1  **Chemotherapy (OPCS-4):**  Check full list in codelist repository file (spreadsheet) | Cancer diagnosis recorded in the 5 years prior to index date AND chemotherapy recorded in 6 months prior to index date | Definition according to the UK Green Book criteria (and the Chief Medical Officer’s recommendations for the shielded-patient list) | <https://assets.publishing.service.gov.uk/government/uploads/system/uploads/attachment_data/file/1057798/Greenbook-chapter-14a-28Feb22.pdf>  https://digital.nhs.uk/coronavirus/shielded-patient-list/methodology/rule-logic |
| Immunosuppression (haematological malignancy or bone marrow transplant) |  | **Haematological malignancy (ICD-10):**  Check full list in codelist repository file (spreadsheet)  **Haematological transplant (ICD-10):**  T86.0  **Haematological transplant (OPCS-4):**  W34, X33.6 | Haematological malignancy or haematological transplant recorded in the 2 years prior to index date |  |  |
| Immunosuppression (solid organ transplant) |  | **Solid organ transplant (ICD-10):**  N16.5, T86.1, T86.2, T86.3, T86.4, T86.8, T86.9, Y83.0, Z94  **Solid organ transplant (OPCS-4):**  Check full list in codelist repository file (spreadsheet) | Solid organ transplant recorded in the 5 years prior to index date |  |  |
| Immunosuppression (hyposplenism) |  | **Hyposplenism (ICD-1):**  D73.0, Q89.0, D57.0, D57.1  **Splenectomy (OPCS-4):**  J69.2 | Hyposplenism or splenectomy recorded in the 5 years prior to index date |  |  |
| Immunosuppression (other long-term) |  | D829, D83, D830, D831, D832,  D838, D839, D84, D840, D841, D848, D849, D71 | Any record within 5 years prior to index date | Manual terminology review and code selection | N/A |
| Immunosuppression (other short-term) |  | **ICD-10:** D70, Y43.3  **OPCS:** X92.1, X95.1 | Any record within 6 months prior to index date |  |  |
| Renal replacement therapy  (peritoneal dialysis) |  | **Peritoneal dialysis (ICD-10):** Z49.2  **Acute kidney injury (ICD-10):** N17  **Peritoneal dialysis (OPCS-4):** X41.1, X40.2, X40.5, X40.6 | Occurrence of any admission with a peritoneal dialysis code (without diagnosis of acute kidney injury) in the 5 years before index date (epistart of the index spell) | Previously-published algorithm | https://www.kidney-international.org/article/S0085-2538(17)30856-6/fulltext |
| Renal replacement therapy  (maintenance haemodialysis) |  | **Dialysis (ICD-10):**  E85.3, Y60.2, Y61.2, Y62.2, Y84.1, Z99.2, T82.4, Z49.1  **Dialysis (OPCS-4):**  X40.1, X40.3, X40.4 | Occurrence of a dialysis code in the 5 years before index date (epistart of the index spell) of the index spell in a patient who has had: |  |  |
|  |  | **ESKD (ICD-10):**  N18.0, N18.5, Q60.1 | (a) a diagnostic code for ESKD any prior time; OR |  |  |
|  |  | **Fistula/graft (OPCS-4):**  L74.1, L74.2, L74.6, L74.8, L74.9 | (b) the insertion of an arteriovenous fistula or graft any prior time. |  |  |
| Renal replacement therapy  (probable maintenance haemodialysis) |  | **Dialysis (ICD-10):**  E85.3, Y60.2, Y61.2, Y62.2, Y84.1, Z99.2, T82.4, Z49.1  **Dialysis (OPCS-4):**  X40.1, X40.3, X40.4 | Occurrence of at least two episodes containing a dialysis code, with at least 90 days between the start of the first recorded dialysis, and the start of any subsequent dialysis (without diagnosis of acute kidney injury) in the 5 years before index date (epistart of the index spell) |  |  |
| Hospital frailty score | diag_01 to diag_20 for ICD-10 codes  (any position); | Codes and scoring used as per reference publication | Last 2 years including index admission | https://www.thelancet.com/journals/lancet/article/PIIS0140-6736(18)30668-8/fulltext | https://www.thelancet.com/journals/lancet/article/PIIS0140-6736(18)30668-8/fulltext |

1. **Geographical location**

We produced a geographical map depicting the ratio (“representativeness ratio”) between relative frequencies of the respective cohort (RECOVERY HES over All-England HES) in each geographical area (based on the lower-super output area/LSOA field in HES). We also included counts of RECOVERY participants recruited in each NHS Trust (based on the Trust’s postcode). The geographical areas presented cover England only and were depicted at England region level.

Mapping from the geographical location recorded in HES (postcode or LSOA) to the geographical areas presented was performed using publicly-available UK government files (available at <https://geoportal.statistics.gov.uk/datasets/postcode-to-output-area-to-lower-layer-super-output-area-to-middle-layer-super-output-area-to-local-authority-district-may-2021-lookup-in-the-uk/about> and <https://geoportal.statistics.gov.uk/datasets/local-authority-district-to-county-april-2021-lookup-in-england/explore>)

1. **Deprivation**

Deprivation was assessed using the aggregated English Index of Multiple Deprivation 2019 (and presented using quintiles). Although HES provides deprivation data in the *imd* fields, these are calculated using the 2004 indices and are therefore no longer up to date. Hence, deprivation will be derived from home residence postcodes included in the RECOVERY HES and national HES records, and mapped using the publicly-available lookup tables at <https://imd-by-postcode.opendatacommunities.org/imd/2019>.

1. **Inclusion in RECOVERY over time**

The total number of individuals included in RECOVERY and the reference population was calculated and aggregated at monthly level (based on the index date). Then, we calculated the monthly proportions of individuals in the Al-England HES cohort who were recruited to RECOVERY (i.e. ratio between absolute counts in each cohort, not on actual matching since the all-England HES cohort is anonymised).

**OUTCOME DEFINITION**

1. **All-cause death within 28 days of admission**

This outcome was only assessed in RECOVERY and the reference population. A timeframe of 28 days from index date was used in both cohorts (rather than 28 days from randomisation in RECOVERY) to allow a more approximate comparison. In both populations, the outcome was derived using HES + Civil Registrations (death record) data only as this is considered the definitive source of mortality records and allowed a meaningful comparison between both cohorts.

**R Studio packages**

Below is a list of all R packages used in this report (generated using the “grateful” package) and an R session info report:

**R packages used**

| Package | Version | Citation |
| --- | --- | --- |
| base | 4.2.1 | R Core Team (2022) |
| beepr | 1.3 | Bååth (2018) |
| broom.helpers | 1.8.0 | Larmarange and Sjoberg (2022) |
| DataExplorer | 0.8.2 | Cui (2020) |
| directlabels | 2021.1.13 | Hocking (2021) |
| flextable | 0.7.2 | Gohel (2022a) |
| forestploter | 0.2.3 | Dayimu (2022) |
| fs | 1.5.2 | Hester, Wickham, and Csárdi (2021) |
| ggh4x | 0.2.3 | van den Brand (2022) |
| ggpattern | 1.0.1 | FC, Davis, and ggplot2 authors (2022) |
| ggrepel | 0.9.1 | Slowikowski (2021) |
| ggvenn | 0.1.9 | Yan (2021) |
| grateful | 0.1.11 | Rodríguez-Sánchez, Jackson, and Hutchins (2022) |
| gtsummary | 1.6.1 | Sjoberg et al. (2021) |
| modelsummary | 1.0.2 | Arel-Bundock (2022) |
| officer | 0.4.3 | Gohel (2022b) |
| patchwork | 1.1.1 | Pedersen (2020) |
| raster | 3.5.21 | Hijmans (2022) |
| RColorBrewer | 1.1.3 | Neuwirth (2022) |
| remotes | 2.4.2 | Csárdi et al. (2021) |
| rgdal | 1.5.32 | Bivand, Keitt, and Rowlingson (2022) |
| scales | 1.2.0 | Wickham and Seidel (2022) |
| tidyverse | 1.3.2 | Wickham et al. (2019) |
| viridis | 0.6.2 | Garnier et al. (2021) |

**Package citations**

Arel-Bundock, Vincent. 2022. “modelsummary: Data and Model Summaries in R.” *Journal of Statistical Software* 103 (1): 1–23. <https://doi.org/10.18637/jss.v103.i01>.

Bååth, Rasmus. 2018. *Beepr: Easily Play Notification Sounds on Any Platform*. <https://CRAN.R-project.org/package=beepr>.

Bivand, Roger, Tim Keitt, and Barry Rowlingson. 2022. *Rgdal: Bindings for the ’Geospatial’ Data Abstraction Library*. <https://CRAN.R-project.org/package=rgdal>.

Csárdi, Gábor, Jim Hester, Hadley Wickham, Winston Chang, Martin Morgan, and Dan Tenenbaum. 2021. *Remotes: R Package Installation from Remote Repositories, Including ’GitHub’*. <https://CRAN.R-project.org/package=remotes>.

Cui, Boxuan. 2020. *DataExplorer: Automate Data Exploration and Treatment*. <https://CRAN.R-project.org/package=DataExplorer>.

Dayimu, Alimu. 2022. *Forestploter: Create Flexible Forest Plot*. <https://github.com/adayim/forestploter>.

FC, Mike, Trevor L Davis, and ggplot2 authors. 2022. *Ggpattern: ’Ggplot2’ Pattern Geoms*. <https://CRAN.R-project.org/package=ggpattern>.

Garnier, Simon, Ross, Noam, Rudis, Robert, Camargo, et al. 2021. *viridis - Colorblind-Friendly Color Maps for r*. <https://doi.org/10.5281/zenodo.4679424>.

Gohel, David. 2022a. *Flextable: Functions for Tabular Reporting*. <https://CRAN.R-project.org/package=flextable>.

———. 2022b. *Officer: Manipulation of Microsoft Word and PowerPoint Documents*. <https://CRAN.R-project.org/package=officer>.

Hester, Jim, Hadley Wickham, and Gábor Csárdi. 2021. *Fs: Cross-Platform File System Operations Based on ’Libuv’*. <https://CRAN.R-project.org/package=fs>.

Hijmans, Robert J. 2022. *Raster: Geographic Data Analysis and Modeling*. <https://CRAN.R-project.org/package=raster>.

Hocking, Toby Dylan. 2021. *Directlabels: Direct Labels for Multicolor Plots*. <https://CRAN.R-project.org/package=directlabels>.

Larmarange, Joseph, and Daniel D. Sjoberg. 2022. *Broom.helpers: Helpers for Model Coefficients Tibbles*. <https://CRAN.R-project.org/package=broom.helpers>.

Neuwirth, Erich. 2022. *RColorBrewer: ColorBrewer Palettes*. <https://CRAN.R-project.org/package=RColorBrewer>.

Pedersen, Thomas Lin. 2020. *Patchwork: The Composer of Plots*. <https://CRAN.R-project.org/package=patchwork>.

R Core Team. 2022. *R: A Language and Environment for Statistical Computing*. Vienna, Austria: R Foundation for Statistical Computing. <https://www.R-project.org/>.

Rodríguez-Sánchez, Francisco, Connor P. Jackson, and Shaurita D. Hutchins. 2022. *Grateful: Facilitate Citation of r Packages*. <https://github.com/Pakillo/grateful>.

Sjoberg, Daniel D., Karissa Whiting, Michael Curry, Jessica A. Lavery, and Joseph Larmarange. 2021. “Reproducible Summary Tables with the Gtsummary Package.” *The R Journal* 13: 570–80. <https://doi.org/10.32614/RJ-2021-053>.

Slowikowski, Kamil. 2021. *Ggrepel: Automatically Position Non-Overlapping Text Labels with ’Ggplot2’*. <https://CRAN.R-project.org/package=ggrepel>.

van den Brand, Teun. 2022. *Ggh4x: Hacks for ’Ggplot2’*. <https://CRAN.R-project.org/package=ggh4x>.

Wickham, Hadley, Mara Averick, Jennifer Bryan, Winston Chang, Lucy D’Agostino McGowan, Romain François, Garrett Grolemund, et al. 2019. “Welcome to the tidyverse.” *Journal of Open Source Software* 4 (43): 1686. <https://doi.org/10.21105/joss.01686>.

Wickham, Hadley, and Dana Seidel. 2022. *Scales: Scale Functions for Visualization*. <https://CRAN.R-project.org/package=scales>.

Yan, Linlin. 2021. *Ggvenn: Draw Venn Diagram by ’Ggplot2’*. <https://CRAN.R-project.org/package=ggvenn>.

**R session info report:**

*session_info()*

*Session info ────────────────────────────────────────────────────────*

*setting value*

*version R version 4.2.1 (2022-06-23 ucrt)*

*os Windows 10 x64 (build 19044)*

*system x86_64, mingw32*

*ui RStudio*

*language (EN)*

*collate English_United Kingdom.utf8*

*ctype English_United Kingdom.utf8*

*tz Europe/London*

*date 2022-11-11*

*rstudio 2022.07.2+576 Spotted Wakerobin (desktop)*

*pandoc 2.19.2 @ C:/Program Files/RStudio/bin/quarto/bin/tools/ (via rmarkdown)*

*─ Packages ────────────────────────────────────────────────────────────*

*package * version date (UTC) lib source*

*assertthat 0.2.1 2019-03-21 [1] CRAN (R 4.2.1)*

*audio 0.1-10 2021-11-25 [1] CRAN (R 4.2.0)*

*backports 1.4.1 2021-12-13 [1] CRAN (R 4.2.0)*

*base64enc 0.1-3 2015-07-28 [1] CRAN (R 4.2.0)*

*beepr * 1.3 2018-06-04 [1] CRAN (R 4.2.1)*

*bit 4.0.4 2020-08-04 [1] CRAN (R 4.2.1)*

*bit64 4.0.5 2020-08-30 [1] CRAN (R 4.2.1)*

*broom 1.0.0 2022-07-01 [1] CRAN (R 4.2.1)*

*broom.helpers 1.8.0 2022-07-05 [1] CRAN (R 4.2.1)*

*cachem 1.0.6 2021-08-19 [1] CRAN (R 4.2.1)*

*callr 3.7.1 2022-07-13 [1] CRAN (R 4.2.1)*

*cellranger 1.1.0 2016-07-27 [1] CRAN (R 4.2.1)*

*checkmate 2.1.0 2022-04-21 [1] CRAN (R 4.2.1)*

*class 7.3-20 2022-01-16 [2] CRAN (R 4.2.1)*

*classInt 0.4-8 2022-09-29 [1] CRAN (R 4.2.1)*

*cli 3.3.0 2022-04-25 [1] CRAN (R 4.2.1)*

*codetools 0.2-18 2020-11-04 [2] CRAN (R 4.2.1)*

*colorspace 2.0-3 2022-02-21 [1] CRAN (R 4.2.1)*

*commonmark 1.8.0 2022-03-09 [1] CRAN (R 4.2.1)*

*crayon 1.5.1 2022-03-26 [1] CRAN (R 4.2.1)*

*data.table 1.14.2 2021-09-27 [1] CRAN (R 4.2.1)*

*DBI 1.1.3 2022-06-18 [1] CRAN (R 4.2.1)*

*dbplyr 2.2.1 2022-06-27 [1] CRAN (R 4.2.1)*

*devtools 2.4.5 2022-10-11 [1] CRAN (R 4.2.2)*

*digest 0.6.29 2021-12-01 [1] CRAN (R 4.2.1)*

*directlabels * 2021.1.13 2021-01-16 [1] CRAN (R 4.2.1)*

*dplyr * 1.0.9 2022-04-28 [1] CRAN (R 4.2.1)*

*dtplyr * 1.2.1 2022-01-19 [1] CRAN (R 4.2.1)*

*e1071 1.7-11 2022-06-07 [1] CRAN (R 4.2.1)*

*ellipsis 0.3.2 2021-04-29 [1] CRAN (R 4.2.1)*

*evaluate 0.15 2022-02-18 [1] CRAN (R 4.2.1)*

*fansi 1.0.3 2022-03-24 [1] CRAN (R 4.2.1)*

*farver 2.1.1 2022-07-06 [1] CRAN (R 4.2.1)*

*fastmap 1.1.0 2021-01-25 [1] CRAN (R 4.2.1)*

*flextable * 0.7.2 2022-06-12 [1] CRAN (R 4.2.1)*

*forcats * 0.5.1 2021-01-27 [1] CRAN (R 4.2.1)*

*forestploter * 0.2.3 2022-11-10 [1] Github (adayim/forestploter@8bf12b2)*

*fs * 1.5.2 2021-12-08 [1] CRAN (R 4.2.1)*

*gargle 1.2.0 2021-07-02 [1] CRAN (R 4.2.1)*

*gdtools 0.2.4 2022-02-14 [1] CRAN (R 4.2.1)*

*generics 0.1.3 2022-07-05 [1] CRAN (R 4.2.1)*

*ggh4x * 0.2.3 2022-11-09 [1] CRAN (R 4.2.2)*

*ggpattern * 1.0.1 2022-11-09 [1] CRAN (R 4.2.2)*

*ggplot2 * 3.4.0 2022-11-04 [1] CRAN (R 4.2.2)*

*ggrepel * 0.9.1 2021-01-15 [1] CRAN (R 4.2.1)*

*ggvenn * 0.1.9 2021-06-29 [1] CRAN (R 4.2.1)*

*glue 1.6.2 2022-02-24 [1] CRAN (R 4.2.1)*

*googledrive 2.0.0 2021-07-08 [1] CRAN (R 4.2.1)*

*googlesheets4 1.0.0 2021-07-21 [1] CRAN (R 4.2.1)*

*gridExtra 2.3 2017-09-09 [1] CRAN (R 4.2.1)*

*gridpattern 1.0.2 2022-11-07 [1] CRAN (R 4.2.2)*

*gt 0.6.0 2022-05-24 [1] CRAN (R 4.2.1)*

*gtable 0.3.1 2022-09-01 [1] CRAN (R 4.2.2)*

*gtsummary * 1.6.1 2022-06-22 [1] CRAN (R 4.2.1)*

*haven * 2.5.0 2022-04-15 [1] CRAN (R 4.2.1)*

*hms 1.1.1 2021-09-26 [1] CRAN (R 4.2.1)*

*htmltools 0.5.3 2022-07-18 [1] CRAN (R 4.2.1)*

*htmlwidgets 1.5.4 2021-09-08 [1] CRAN (R 4.2.1)*

*httpuv 1.6.5 2022-01-05 [1] CRAN (R 4.2.1)*

*httr 1.4.3 2022-05-04 [1] CRAN (R 4.2.1)*

*jsonlite 1.8.0 2022-02-22 [1] CRAN (R 4.2.1)*

*KernSmooth 2.23-20 2021-05-03 [2] CRAN (R 4.2.1)*

*knitr 1.39 2022-04-26 [1] CRAN (R 4.2.1)*

*labeling 0.4.2 2020-10-20 [1] CRAN (R 4.2.0)*

*later 1.3.0 2021-08-18 [1] CRAN (R 4.2.1)*

*lattice 0.20-45 2021-09-22 [2] CRAN (R 4.2.1)*

*lifecycle 1.0.3 2022-10-07 [1] CRAN (R 4.2.2)*

*lubridate * 1.8.0 2021-10-07 [1] CRAN (R 4.2.1)*

*magrittr * 2.0.3 2022-03-30 [1] CRAN (R 4.2.1)*

*memoise 2.0.1 2021-11-26 [1] CRAN (R 4.2.1)*

*mime 0.12 2021-09-28 [1] CRAN (R 4.2.0)*

*miniUI 0.1.1.1 2018-05-18 [1] CRAN (R 4.2.1)*

*modelr 0.1.8 2020-05-19 [1] CRAN (R 4.2.1)*

*modelsummary * 1.0.2 2022-07-17 [1] CRAN (R 4.2.1)*

*munsell 0.5.0 2018-06-12 [1] CRAN (R 4.2.1)*

*officer * 0.4.3 2022-06-12 [1] CRAN (R 4.2.1)*

*patchwork * 1.1.1 2020-12-17 [1] CRAN (R 4.2.1)*

*pillar 1.8.0 2022-07-18 [1] CRAN (R 4.2.1)*

*pkgbuild 1.3.1 2021-12-20 [1] CRAN (R 4.2.2)*

*pkgconfig 2.0.3 2019-09-22 [1] CRAN (R 4.2.1)*

*pkgload 1.3.0 2022-06-27 [1] CRAN (R 4.2.1)*

*prettyunits 1.1.1 2020-01-24 [1] CRAN (R 4.2.1)*

*processx 3.7.0 2022-07-07 [1] CRAN (R 4.2.1)*

*profvis 0.3.7 2020-11-02 [1] CRAN (R 4.2.2)*

*promises 1.2.0.1 2021-02-11 [1] CRAN (R 4.2.1)*

*proxy 0.4-27 2022-06-09 [1] CRAN (R 4.2.1)*

*ps 1.7.1 2022-06-18 [1] CRAN (R 4.2.1)*

*purrr * 0.3.4 2020-04-17 [1] CRAN (R 4.2.1)*

*quadprog 1.5-8 2019-11-20 [1] CRAN (R 4.2.0)*

*R6 2.5.1 2021-08-19 [1] CRAN (R 4.2.1)*

*ragg 1.2.4 2022-10-24 [1] CRAN (R 4.2.2)*

*raster 3.5-21 2022-06-27 [1] CRAN (R 4.2.1)*

*RColorBrewer * 1.1-3 2022-04-03 [1] CRAN (R 4.2.0)*

*Rcpp 1.0.9 2022-07-08 [1] CRAN (R 4.2.1)*

*readr * 2.1.2 2022-01-30 [1] CRAN (R 4.2.1)*

*readxl * 1.4.0 2022-03-28 [1] CRAN (R 4.2.1)*

*remotes 2.4.2 2021-11-30 [1] CRAN (R 4.2.1)*

*reprex 2.0.1 2021-08-05 [1] CRAN (R 4.2.1)*

*rgdal * 1.5-32 2022-05-09 [1] CRAN (R 4.2.1)*

*rlang 1.0.6 2022-09-24 [1] CRAN (R 4.2.2)*

*rmarkdown 2.14 2022-04-25 [1] CRAN (R 4.2.1)*

*rstudioapi 0.13 2020-11-12 [1] CRAN (R 4.2.1)*

*rvest 1.0.2 2021-10-16 [1] CRAN (R 4.2.1)*

*sass 0.4.2 2022-07-16 [1] CRAN (R 4.2.1)*

*scales * 1.2.0 2022-04-13 [1] CRAN (R 4.2.1)*

*sessioninfo 1.2.2 2021-12-06 [1] CRAN (R 4.2.2)*

*sf 1.0-8 2022-07-14 [1] CRAN (R 4.2.1)*

*shiny 1.7.2 2022-07-19 [1] CRAN (R 4.2.1)*

*sp * 1.5-0 2022-06-05 [1] CRAN (R 4.2.1)*

*stringi 1.7.8 2022-07-11 [1] CRAN (R 4.2.1)*

*stringr * 1.4.0 2019-02-10 [1] CRAN (R 4.2.1)*

*systemfonts 1.0.4 2022-02-11 [1] CRAN (R 4.2.1)*

*tables 0.9.6 2020-09-22 [1] CRAN (R 4.2.1)*

*terra 1.6-3 2022-07-25 [1] CRAN (R 4.2.1)*

*textshaping 0.3.6 2021-10-13 [1] CRAN (R 4.2.2)*

*tibble * 3.1.8 2022-07-22 [1] CRAN (R 4.2.1)*

*tidyr * 1.2.0 2022-02-01 [1] CRAN (R 4.2.1)*

*tidyselect 1.1.2 2022-02-21 [1] CRAN (R 4.2.1)*

*tidyverse * 1.3.2 2022-07-18 [1] CRAN (R 4.2.1)*

*tzdb 0.3.0 2022-03-28 [1] CRAN (R 4.2.1)*

*units 0.8-0 2022-02-05 [1] CRAN (R 4.2.1)*

*urlchecker 1.0.1 2021-11-30 [1] CRAN (R 4.2.2)*

*usethis 2.1.6 2022-05-25 [1] CRAN (R 4.2.2)*

*utf8 1.2.2 2021-07-24 [1] CRAN (R 4.2.1)*

*uuid 1.1-0 2022-04-19 [1] CRAN (R 4.2.0)*

*vctrs 0.5.0 2022-10-22 [1] CRAN (R 4.2.2)*

*viridis * 0.6.2 2021-10-13 [1] CRAN (R 4.2.1)*

*viridisLite * 0.4.0 2021-04-13 [1] CRAN (R 4.2.1)*

*vroom 1.5.7 2021-11-30 [1] CRAN (R 4.2.1)*

*withr 2.5.0 2022-03-03 [1] CRAN (R 4.2.1)*

*xfun 0.31 2022-05-10 [1] CRAN (R 4.2.1)*

*xml2 1.3.3 2021-11-30 [1] CRAN (R 4.2.1)*

*xtable 1.8-4 2019-04-21 [1] CRAN (R 4.2.1)*

*zip 2.2.0 2021-05-31 [1] CRAN (R 4.2.1)*

**References (Annex I)**

1. UK Health Security Agency. COVID-19: the green book, chapter 14a. 2022.

2. Storey BC, Staplin N, Harper CH, et al. Declining comorbidity-adjusted mortality rates in English patients receiving maintenance renal replacement therapy. *Kidney Int*. 2018 May;**93**(5):1165–1174.

3. Quan H, Sundararajan V, Halfon P, et al. Coding Algorithms for Defining Comorbidities in ICD-9-CM and ICD-10 Administrative Data: *Med Care*. 2005 Nov;**43**(11):1130–1139.

4. Gilbert T, Neuburger J, Kraindler J, et al. Development and validation of a Hospital Frailty Risk Score focusing on older people in acute care settings using electronic hospital records: an observational study. *Lancet Lond Engl*. 2018 May 5;**391**(10132):1775–1782.

5. Centers for Disease Control and Prevention. Alcohol-Related ICD Codes [Internet]. 2021 [cited 2022 Oct 11]. Available from: https://www.cdc.gov/alcohol/ardi/alcohol-related-icd-codes.html

6. Khokhar B, Jette N, Metcalfe A, et al. Systematic review of validated case definitions for diabetes in ICD-9-coded and ICD-10-coded data in adult populations. *BMJ Open*. 2016 Aug;**6**(8):e009952.

7. NHS Digital. NHS Data Model and Dictionary - Severe Mental Illness [Internet]. 2022 [cited 2022 Oct 11]. Available from: https://www.datadictionary.nhs.uk/Covid19PRA/Severe_Mental_Illness.html

8. Gribsholt SB, Pedersen L, Richelsen B, Thomsen RW. Validity of ICD-10 diagnoses of overweight and obesity in Danish hospitals. *Clin Epidemiol*. 2019;**11**:845–854.

## Annex II – Supplementary tables and figures

### Supplementary table S1 - Baseline characteristics of the RECOVERY population recruited in the UK, grouped by nation (using data from the case report form only, except where stated)

| Characteristic | England, N = 39952^a^ | Northern Ireland, N = 985 ^a^ | Scotland, N = 2496 ^a^ | Wales, N = 1333 ^a^ |
| --- | --- | --- | --- | --- |
| Age, mean (SD) | 62 (16) | 58 (15) | 62 (15) | 61 (15) |
| <60 | 17063 (43%) | 505 (51%) | 1036 (42%) | 602 (45%) |
| 60-69 | 9154 (23%) | 256 (26%) | 657 (26%) | 350 (26%) |
| 70-79 | 8047 (20%) | 156 (16%) | 473 (19%) | 257 (19%) |
| 80-89 | 4709 (12%) | 60 (6.1%) | 268 (11%) | 105 (7.9%) |
| 90+ | 979 (2.5%) | 8 (0.8%) | 62 (2.5%) | 19 (1.4%) |
| Female | 14616 (37%) | 340 (35%) | 958 (38%) | 487 (37%) |
| Ethnicity^b^ |  |  |  |  |
| White | 32205 (82%) | N/A | 2040 (97%) | 574 (93%) |
| Black | 1491 (3.8%) | N/A | 13 (0.6%) | 3 (0.5%) |
| Asian | 3964 (10%) | N/A | 44 (2.1%) | 23 (3.7%) |
| Other | 948 (2.4%) | N/A | 7 (0.3%) | 8 (1.3%) |
| Mixed | 516 (1.3%) | N/A | 5 (0.2%) | 6 (1.0%) |
| Unknown | 828 (2.1%) | N/A | 387 (16%) | 719 (54%) |
| Respiratory support status^c^ |  |  |  |  |
| Invasive mechanical ventilation or ECMO | 2674 (6.7%) | 46 (4.7%) | 180 (7.2%) | 233 (17%) |
| Non-invasive mechanical ventilation or supplementary oxygen | 32420 (81%) | 851 (86%) | 1955 (78%) | 913 (68%) |
| None | 4858 (12%) | 88 (8.9%) | 361 (14%) | 187 (14%) |
| Chronic lung disease | 8784 (22%) | 200 (20%) | 572 (23%) | 290 (22%) |
| Diabetes | 10434 (26%) | 209 (21%) | 564 (23%) | 313 (23%) |
| Chronic heart disease | 9120 (23%) | 174 (18%) | 506 (20%) | 250 (19%) |
| Severe liver disease | 471 (1.2%) | 10 (1.0%) | 33 (1.3%) | 19 (1.4%) |
| Severe renal impairment | 2238 (5.6%) | 19 (1.9%) | 76 (3.0%) | 76 (5.7%) |
| Cohorts restricted to participants randomised before the 1^st^ January 2022, with no age restrictions. ECMO - extracorporeal membrane oxygenation; HES - Hospital Episode Statistics; SD – standard deviation; | | | | |
| ^a^Mean (SD); n (%) | | | | |
| ^b^Ethnicity extracted from either primary care data (General Practice Extraction Service Data for Pandemic Planning and Research - GDPPR) or HES data for people in England, and data sources equivalent to HES Scotland and Wales; no such data was available in Northern Ireland; proportions for people with known and unknown ethnicity were calculated separately and using the entire cohort as denominator | | | | |
| ^c^Respiratory status derived from the case-report form combined with linked data sources such as hospital admissions and intensive care data (except for Northern Ireland where no linkage data was available) | | | | |

### Supplementary table S2 - Baseline characteristics of the RECOVERY population recruited in England, grouped by HES linkage status (using data from the case report form only, except where stated)

| Characteristic | HES data available,  N = 38510^a^ | HES data unavailable,  N = 648^a^ |
| --- | --- | --- |
| Age, mean (SD) | 63 (15) | 58 (15) |
| <60 | 16121 (42%) | 349 (54%) |
| 60-69 | 8906 (23%) | 146 (23%) |
| 70-79 | 7872 (20%) | 107 (17%) |
| 80-89 | 4644 (12%) | 37 (5.7%) |
| 90+ | 967 (2.5%) | 9 (1.4%) |
| Female | 14068 (37%) | 245 (38%) |
| Ethnicity^2^ |  |  |
| White | 31326 (83%) | 330 (64%) |
| Black | 1367 (3.6%) | 71 (14%) |
| Asian | 3809 (10%) | 67 (13%) |
| Other | 877 (2.3%) | 25 (4.9%) |
| Mixed | 465 (1.2%) | 19 (3.7%) |
| Unknown | 666 (1.7%) | 136 (21%) |
| Respiratory support status^3^ |  |  |
| Invasive mechanical ventilation or ECMO | 2562 (6.7%) | 72 (11%) |
| Non-invasive mechanical ventilation or supplementary oxygen | 31363 (81%) | 504 (78%) |
| None | 4585 (12%) | 72 (11%) |
| Chronic lung disease | 8569 (22%) | 130 (20%) |
| Diabetes | 10193 (26%) | 152 (23%) |
| Chronic heart disease | 8936 (23%) | 108 (17%) |
| Severe liver disease | 448 (1.2%) | 19 (2.9%) |
| Severe renal impairment | 2185 (5.7%) | 39 (6.0%) |
| Restricted to RECOVERY participants aged >=16 years and recruited in England within the analysis period. ECMO - extracorporeal membrane oxygenation; HES - Hospital Episode Statistics | | |
| ^a^Mean (SD); n (%) | | |
| ^b^Ethnicity extracted from either primary care data (General Practice Extraction Service Data for Pandemic Planning and Research - GDPPR) or HES data. Proportions for people with known and unknown ethnicity were calculated separately, using the entire cohort as denominator | | |
| ^c^Respiratory status derived from the case-report form combined with linked data sources such as hospital admissions and intensive care data | | |

### Supplementary table S3 - Number of individuals included in RECOVERY versus reference population over time, by age groups

| Period | Age bands | | | | | | | | | | | | Aggregate | | | |
| --- | --- | --- | --- | --- | --- | --- | --- | --- | --- | --- | --- | --- | --- | --- | --- | --- |
|  | <60 | | | 60-69 | | | 70-79 | | | 80+ | | |  |  |  |  |
|  | RECOVERY (% of all ages total) | Reference population (% of all ages total) | Proportion included in RECOVERY (%) | RECOVERY (% of all ages total) | Reference population (% of all ages total) | Proportion included in RECOVERY (%) | RECOVERY (% of all ages total) | Reference population (% of all ages total) | Proportion included in RECOVERY (%) | RECOVERY (% of all ages total) | Reference population (% of all ages total) | Proportion included in RECOVERY (%) | RECOVERY (% of all ages total) | Reference population (% of all ages total) | Proportion included in RECOVERY (%) |  |
| All time periods | 16121 (100) | 123790 (100) | 13 | 8906 (100) | 56452 (100) | 15.8 | 7871 (100) | 69107 (100) | 11.4 | 5612 (100) | 96922 (100) | 5.8 | 38510 | 346271 | 11.1 |  |
| Mar 20 - May 20 | 3337 (20.7) | 21514 (17.4) | 15.5 | 1992 (22.4) | 12082 (21.4) | 16.5 | 2068 (26.3) | 16915 (24.5) | 12.2 | 2237 (39.9) | 27669 (28.5) | 8.1 | 9634 | 78180 | 12.3 |  |
| Jun 20 - Aug 20 | 311 (1.9) | 2095 (1.7) | 14.8 | 188 (2.1) | 1084 (1.9) | 17.3 | 218 (2.8) | 1396 (2) | 15.6 | 285 (5.1) | 2364 (2.4) | 12.1 | 1002 | 6939 | 14.4 |  |
| Sep 20 - Nov 20 | 2263 (14) | 14494 (11.7) | 15.6 | 1512 (17) | 8082 (14.3) | 18.7 | 1488 (18.9) | 11252 (16.3) | 13.2 | 945 (16.8) | 15603 (16.1) | 6.1 | 6208 | 49431 | 12.6 |  |
| Dec 20 - Feb 21 | 7280 (45.2) | 43526 (35.2) | 16.7 | 4143 (46.5) | 22371 (39.6) | 18.5 | 3241 (41.2) | 25535 (36.9) | 12.7 | 1784 (31.8) | 36318 (37.5) | 4.9 | 16448 | 127750 | 12.9 |  |
| Mar 21 - May 21 | 716 (4.4) | 5129 (4.1) | 14 | 264 (3) | 1488 (2.6) | 17.7 | 93 (1.2) | 1086 (1.6) | 8.6 | 87 (1.6) | 1616 (1.7) | 5.4 | 1160 | 9319 | 12.4 |  |
| Jun 21 - Aug 21 | 1162 (7.2) | 19016 (15.4) | 6.1 | 321 (3.6) | 4266 (7.6) | 7.5 | 313 (4) | 4453 (6.4) | 7 | 109 (1.9) | 4729 (4.9) | 2.3 | 1905 | 32464 | 5.9 |  |
| Sep 21 - Nov 21 | 1052 (6.5) | 18016 (14.6) | 5.8 | 486 (5.5) | 7079 (12.5) | 6.9 | 450 (5.7) | 8470 (12.3) | 5.3 | 165 (2.9) | 8623 (8.9) | 1.9 | 2153 | 42188 | 5.1 |  |

### Supplementary table S4 - 28-day mortality along time in the RECOVERY HES and All-England HES cohorts (split by age groups)

| Period |  | Age bands | | | | | | | | | | | | | | | |  |
| --- | --- | --- | --- | --- | --- | --- | --- | --- | --- | --- | --- | --- | --- | --- | --- | --- | --- | --- |
|  | <60 | | | | 60-69 | | | | 70-79 | | | | | 80+ | | | |  |
|  | RECOVERY | | Reference population | | RECOVERY | | Reference population | | RECOVERY | | Reference population | | | RECOVERY | | Reference population | |  |
|  | Deaths/  Population | Mortality (%) | Deaths/  Population | Mortality (%) | Deaths/  Population | Mortality (%) | Deaths/  Population | Mortality (%) | Deaths/  Population | Mortality (%) | Deaths/  Population | Mortality (%) | Deaths/  Population | | Mortality (%) | Deaths/  Population | Mortality (%) | |
| All time periods | 1144/  16109 | 7.1 | 6901/  123429 | 5.6 | 1789/  8897 | 20.1 | 11031/  56435 | 19.5 | 2584/  7868 | 32.8 | 22481/  69097 | 32.5 | 2400/  5610 | | 42.8 | 45296/  96665 | 46.9 | |
| Mar 20 - May 20 | 309/  3333 | 9.3 | 2103/  21490 | 9.8 | 465/  1990 | 23.4 | 3250/  12077 | 26.9 | 681/  2067 | 32.9 | 6925/  16914 | 40.9 | 907/  2236 | | 40.6 | 14574/  27665 | 52.7 | |
| Jun 20 - Aug 20 | 14/  311 | 4.5 | 105/  2076 | 5.1 | 37/  188 | 19.7 | 182/  1084 | 16.8 | 68/  217 | 31.3 | 387/  1395 | 27.7 | 118/  285 | | 41.4 | 896/  2364 | 37.9 | |
| Sep 20 - Nov 20 | 133/  2260 | 5.9 | 710/  14492 | 4.9 | 291/  1510 | 19.3 | 1438/  8079 | 17.8 | 505/  1488 | 33.9 | 3481/  11252 | 30.9 | 404/  945 | | 42.8 | 6925/  15600 | 44.4 | |
| Dec 20 - Feb 21 | 547/  7276 | 7.5 | 2538/  43512 | 5.8 | 826/  4138 | 20.0 | 4342/  22364 | 19.4 | 1115/  3240 | 34.4 | 8602/  25528 | 33.7 | 841/  1783 | | 47.2 | 17871/  36316 | 49.2 | |
| Mar 21 - May 21 | 22/  716 | 3.1 | 127/  5128 | 2.5 | 39/  264 | 14.8 | 192/  1488 | 12.9 | 22/  93 | 23.7 | 280/  1086 | 25.8 | 42/  87 | | 48.3 | 617/  1542 | 40.0 | |
| Jun 21 - Aug 21 | 60/  1161 | 5.2 | 612/  19012 | 3.2 | 64/  321 | 19.9 | 607/  4266 | 14.2 | 82/  313 | 26.2 | 1021/  4452 | 22.9 | 42/  109 | | 38.5 | 1521/  4555 | 33.4 | |
| Sep 21 - Nov 21 | 59/  1052 | 5.6 | 706/  17719 | 4.0 | 67/  486 | 13.8 | 1020/  7077 | 14.4 | 111/  450 | 24.7 | 1785/  8470 | 21.1 | 46/  165 | | 27.9 | 2892/  8623 | 33.5 | |

### Supplementary figure S1 - Age and sex representativeness of RECOVERY in comparison with the reference population


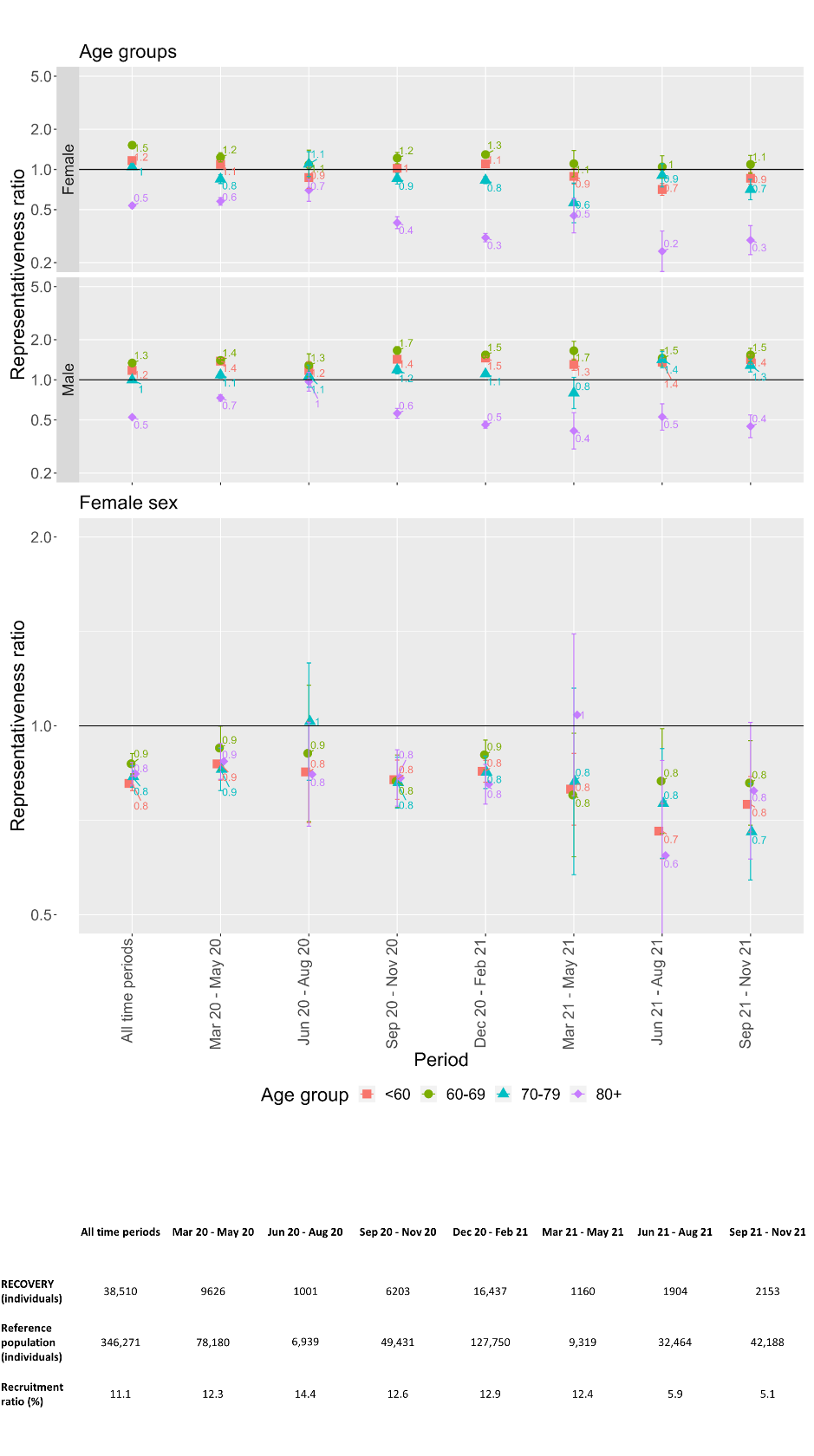


Vertical axes plotted using a log2 scale. Representativeness ratio calculated as the ratio between the proportion of individuals in each age group in RECOVERY over the reference population (i.e. ratio > 1 indicates overrepresentation in RECOVERY, ratio <1 indicates underrepresentation). Error bars plotted using 95% confidence intervals.

###

### Supplementary figure S2 - Ethnicity over time in the RECOVERY cohort in comparison with the reference population, by age


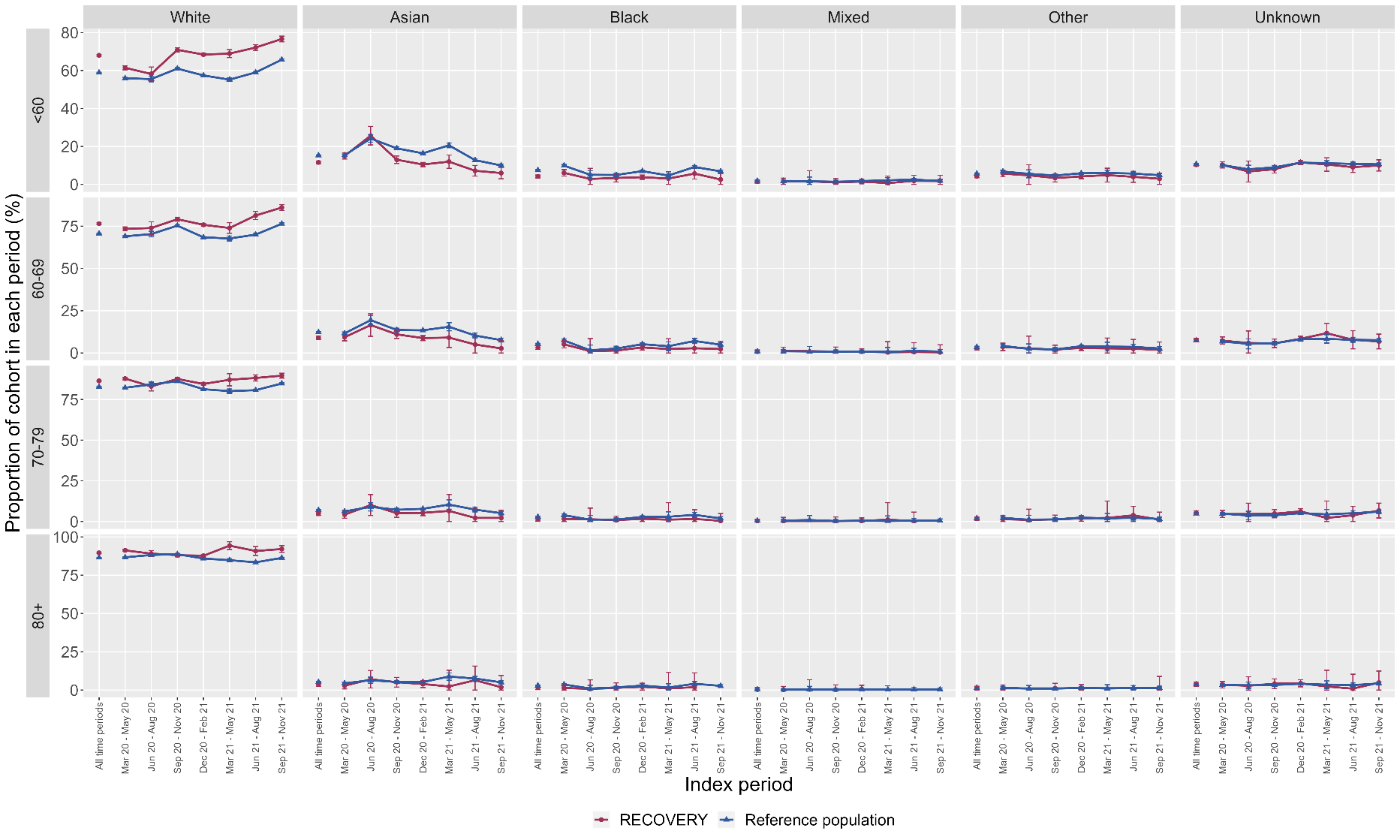


Error bars depict standard errors

### Supplementary figure S3 - Deprivation over time in the RECOVERY cohort in comparison with the reference population, by age


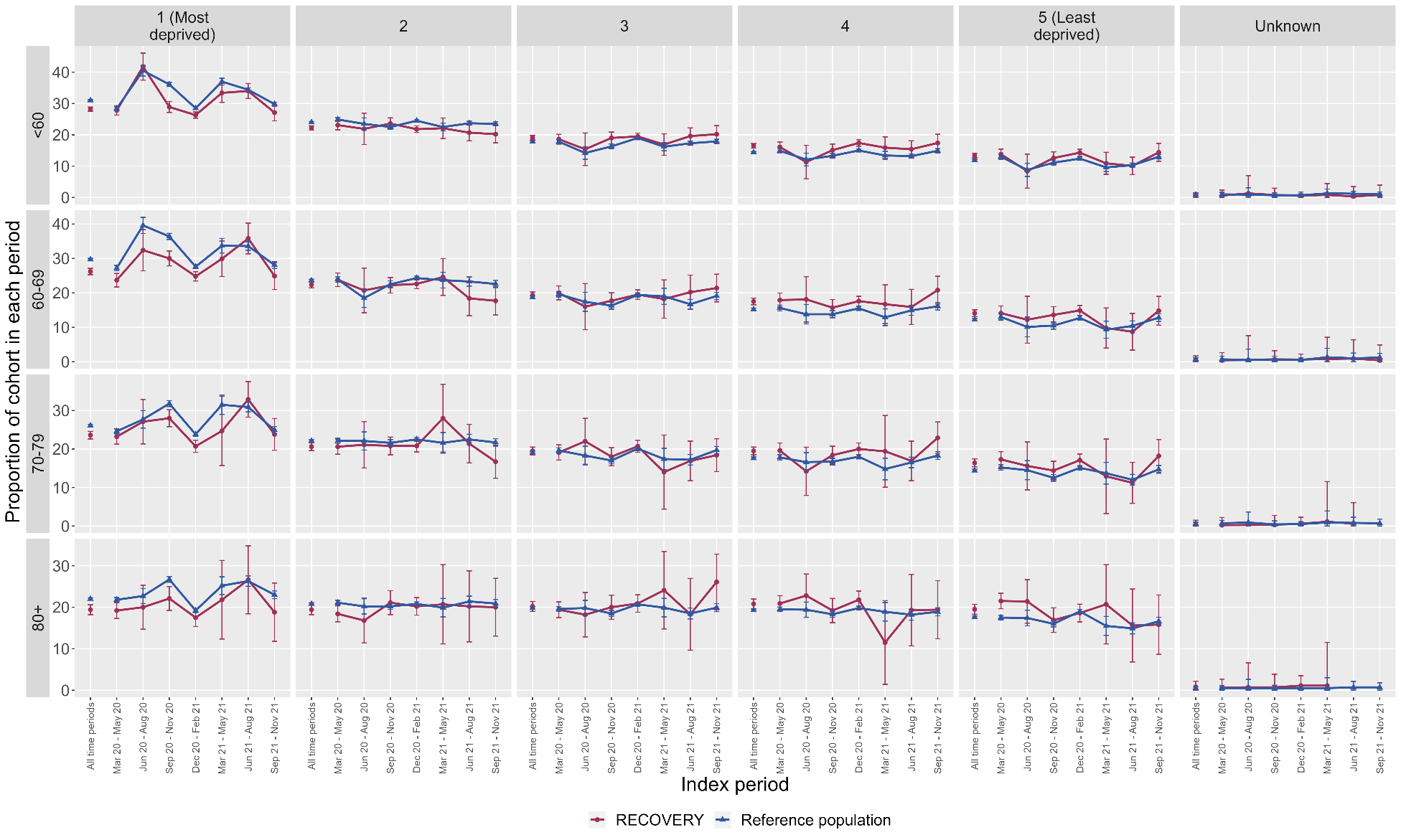


Error bars depict standard errors

###

### Supplementary figure S4 - Charlson Comorbidty Score (excluding age) and Hospital Frailty Risk Score in the RECOVERY cohort and the reference population, by age groups


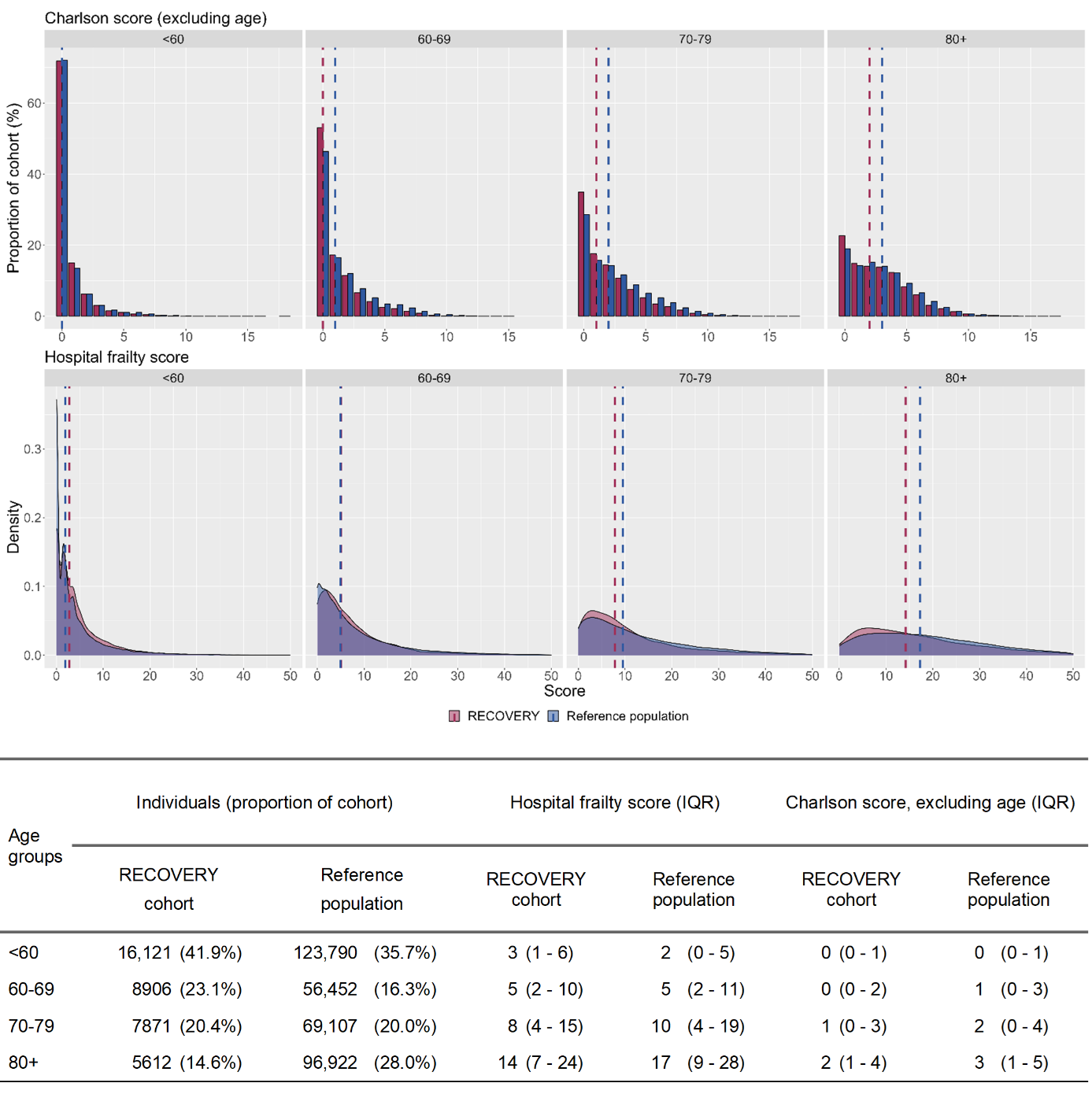


Vertical dashed bars depict the median in each cohort

### Supplementary figure S5 - Hospital Frailty Risk Score over time in the RECOVERY cohort and the reference population, by age groups


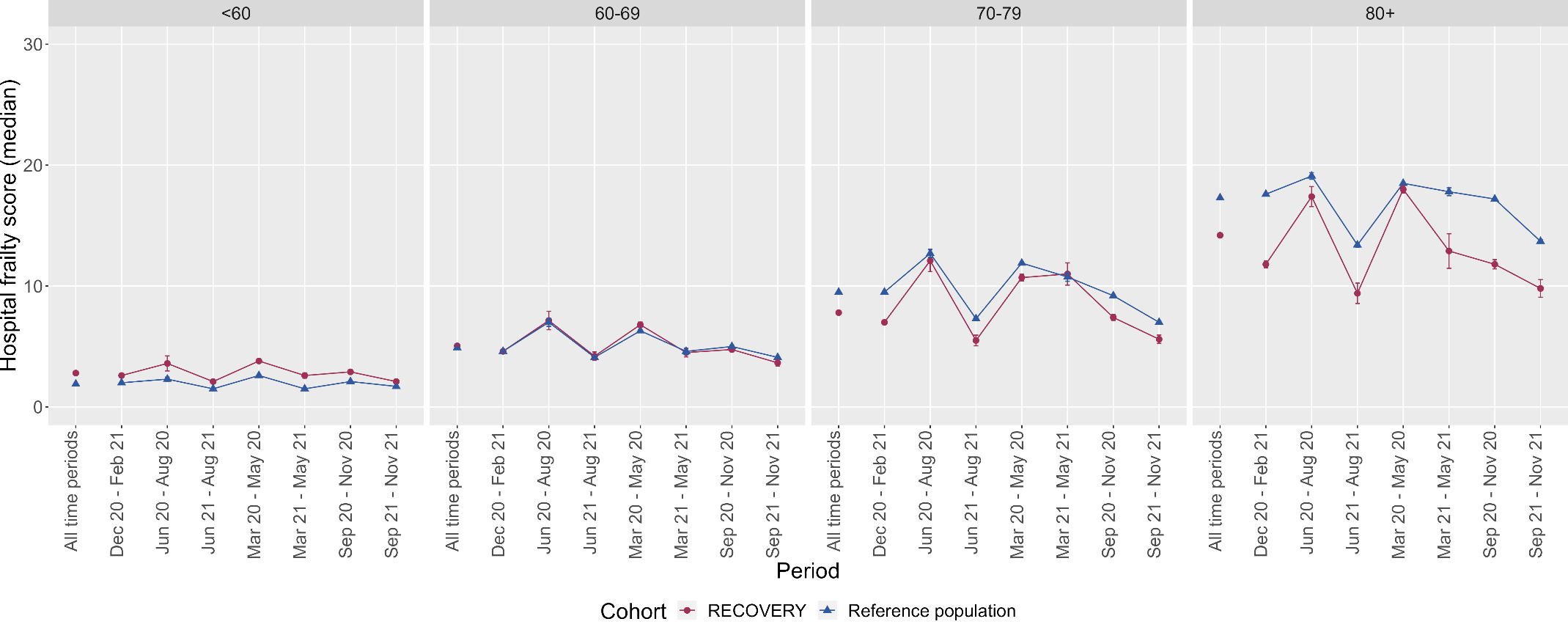


Error bars depict standard errors

### Supplementary figure S6 - Prevalence of select comorbidities in the RECOVERY cohort and reference population, by age groups


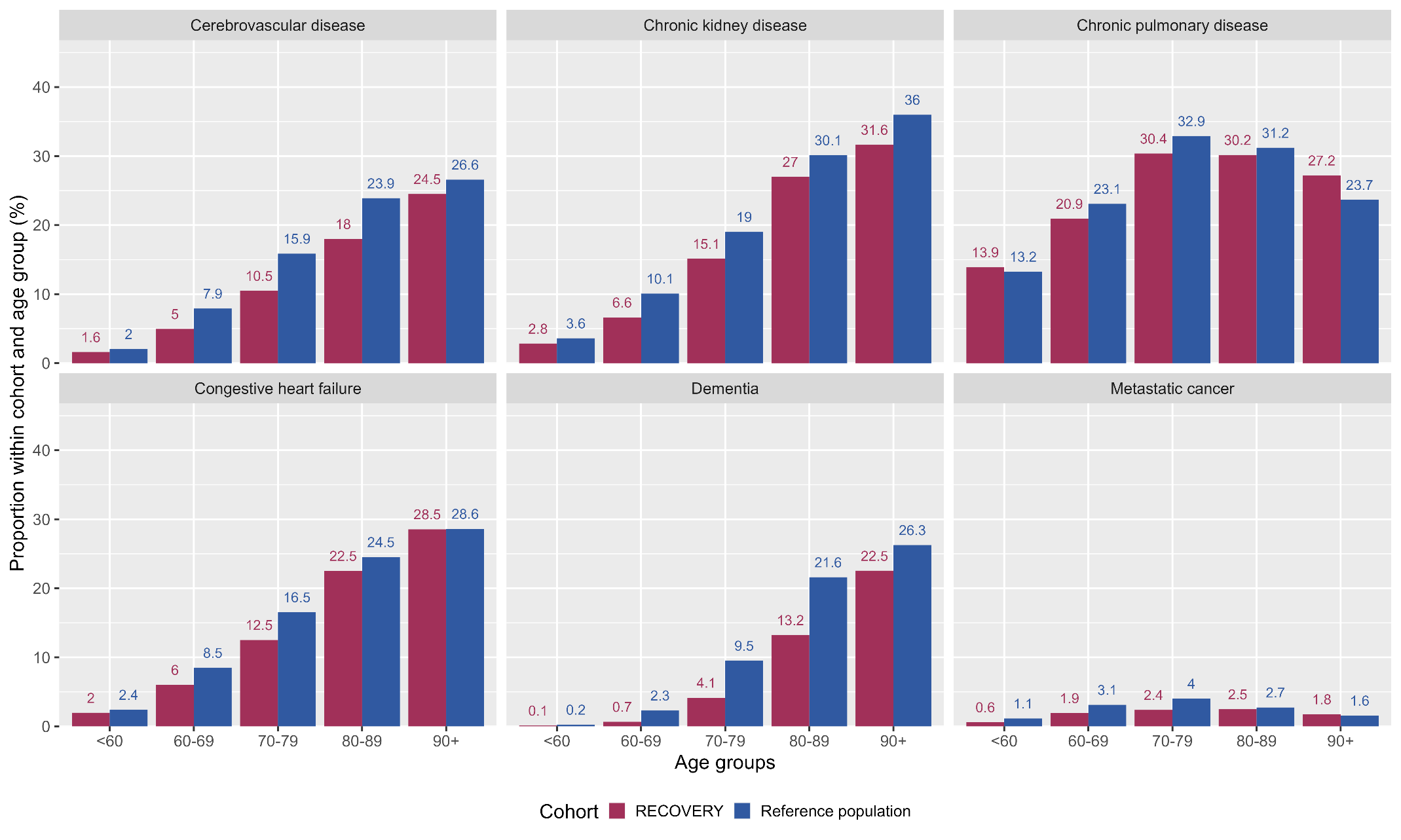


### Supplementary figure S7 - 28-day mortality in the RECOVERY HES and All-England HES populations (stratified by age and sex)


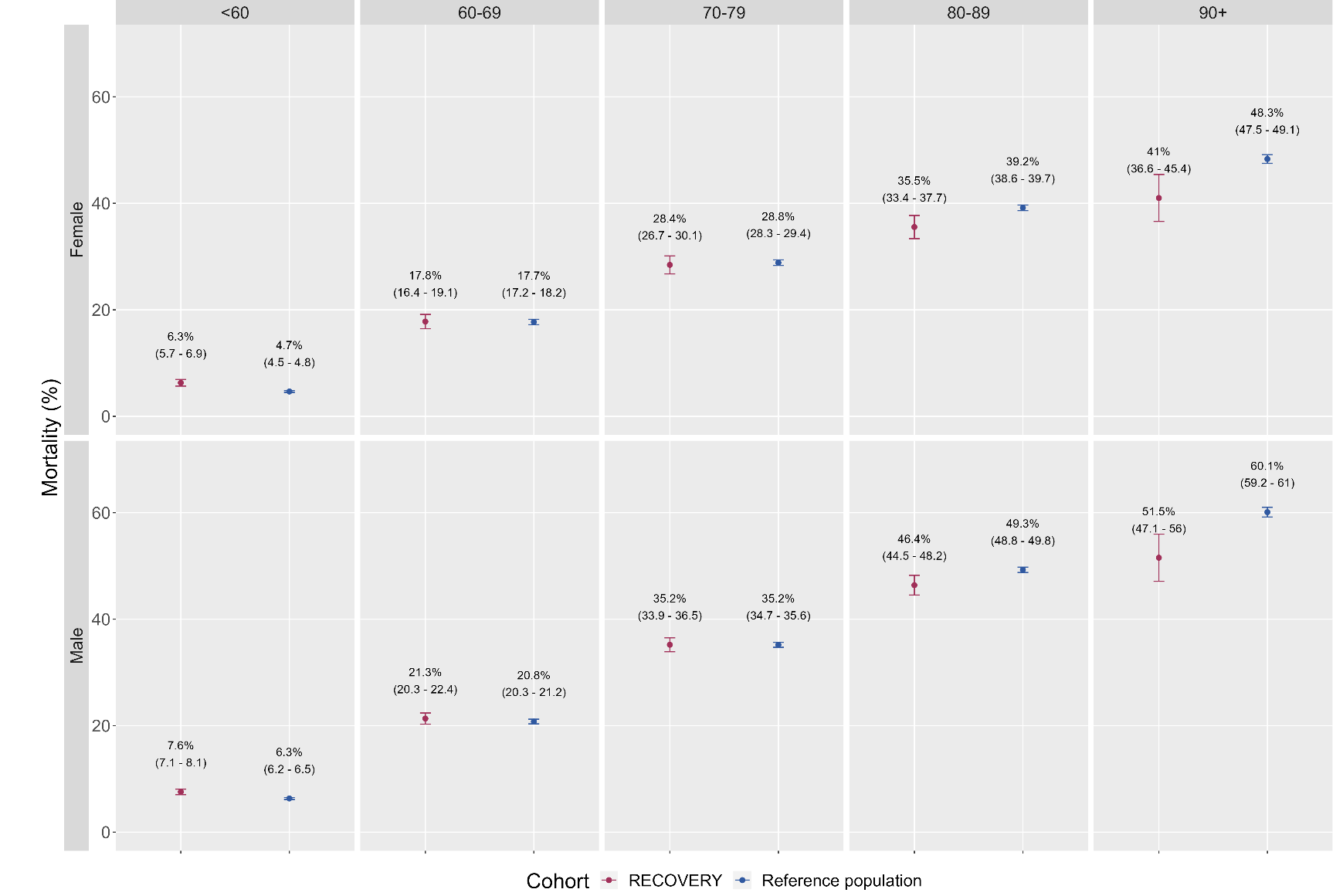


Error bars show 95% confidence intervals

### Supplementary figure S8 - 28-day mortality over time in RECOVERY and the reference population (by age groups)


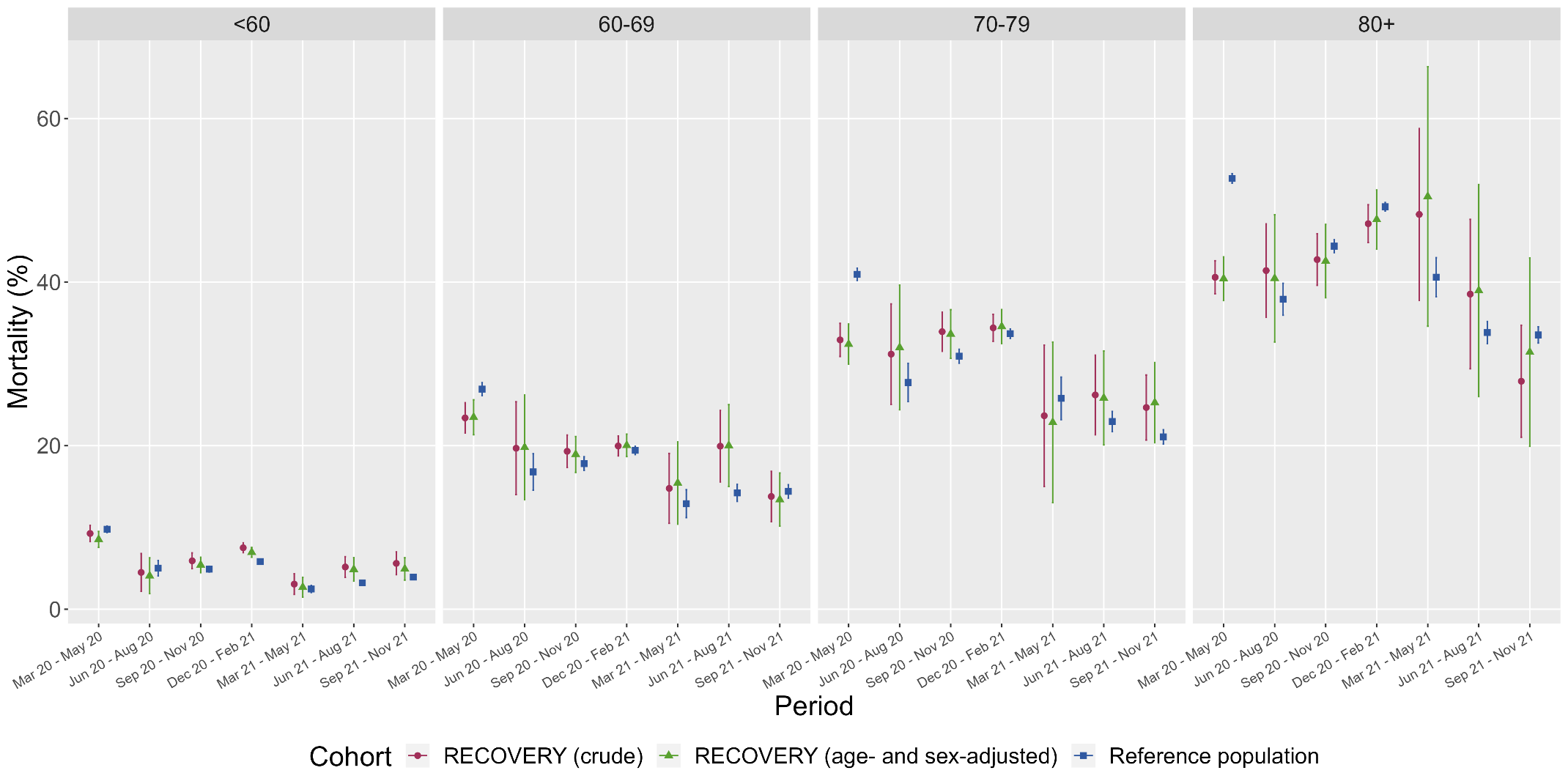


28-day mortality presented as the proportion of people with death recorded within 28 days of their index date along with 95% confidence intervals.

### Supplementary figure S9 - Mortality rates over time in RECOVERY and the reference population, by age, comorbidity, and frailty


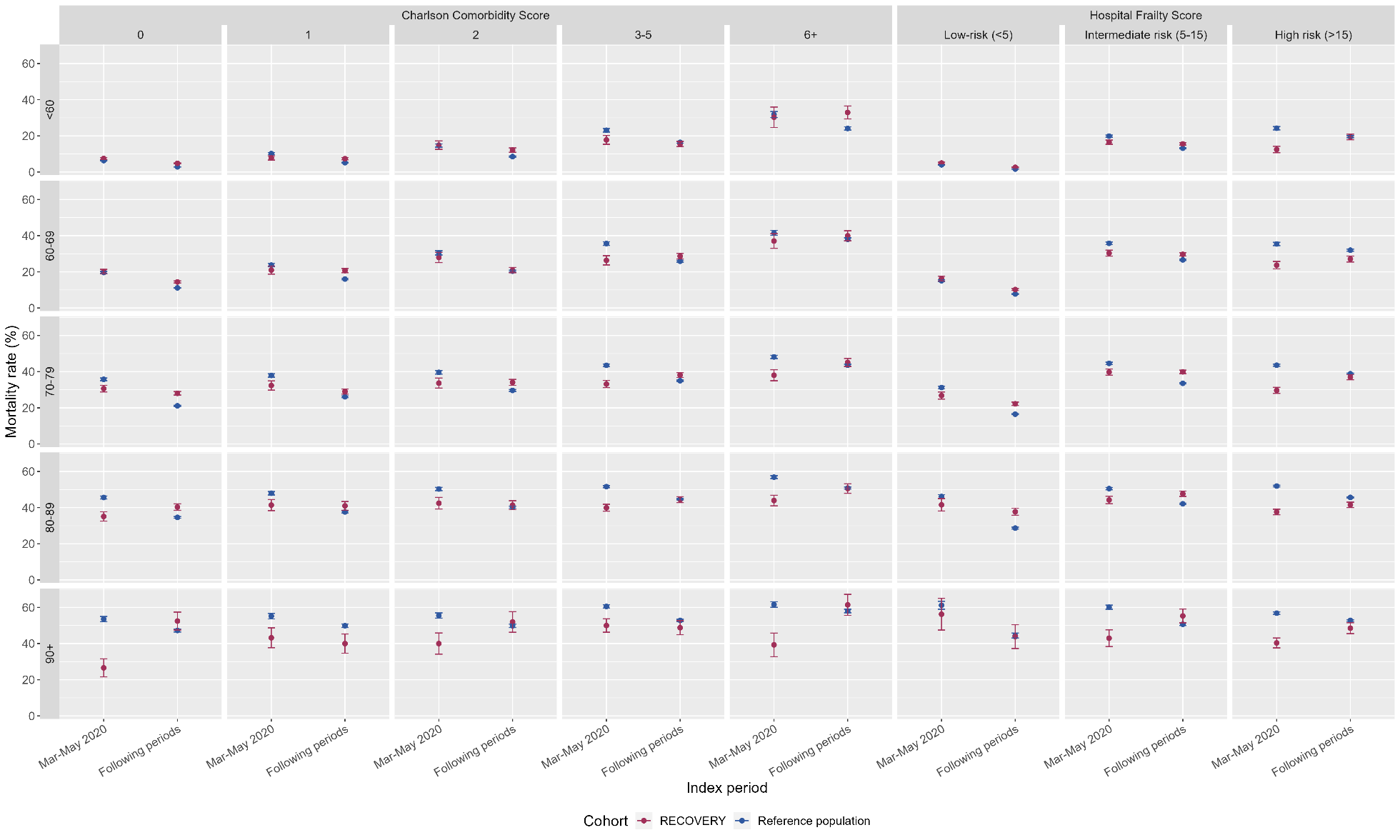


Error bars depict standard errors

## Annex III - Cross-coding of COVID-19 coding in Hospital Episode Statistics versus testing data in SGSS within the RECOVERY population

For this project, a wealth of linked datasets was available for the RECOVERY population (including admissions data from Hospital Episode Statistics – HES, and in-hospital COVID-19 testing from Second Generation Surveillance System data - SGSS). However, for the reference population (anonymised All-England cohort), only HES/ONS data were available. Therefore, preliminary validation work was undertaken to validate the proposed approach of building a nationwide cohort of patients admitted to hospital with COVID-19 using HES data only by exploring cross-coding of ICD-10 codes (in HES) and SARS-CoV-2 testing (from the SGSS data) in the RECOVERY population (which may be seen as a population with a proven or suspected clinical diagnosis of COVID-19). This analysis was based on RECOVERY participants recruited in England between March 2020 and November 2021, using HES data received in September 2022.

1. **COVID-19 coding in HES (any code) vs SGSS testing records**

Of 38920 RECOVERY participants with HES data available:

1. 98.7% (n=38412) had a HES spell straddling randomisation date;
2. 96.1% (n=37047) had a HES spell containing COVID-19 codes in any diagnostic position at any time point, and 91.2% (n=35493) had a HES spell containing COVID-19 codes in the primary diagnostic position at any time point;
3. 94.8% (n=36943) had a HES spell straddling randomisation date and containing COVID-19 codes in any diagnostic position;
4. 89.7% (n=34929) had a HES spell straddling randomisation date and containing COVID-19 codes in the primary diagnostic position; of these, 93.8% (n=32770) had a positive test recorded in SGSS, and 6.2% (n=2159) did not;
5. 91.5% (n=35614) had a positive test recorded in SGSS - of these, 92.0% (n=32770) had a COVID-19 code in the primary diagnostic position in the HES record straddling randomisation date, and 8.0% (n=2844) did not;
6. 8.7% (n=3391) had no COVID-19 codes in the primary diagnostic position in the HES record straddling randomisation – of these, 63.7% (n=2159) had a positive test recorded in SGSS, and 36.3% (n=1232) did not;
7. 8.5% (n=3306) had no SGSS records – of these, 71.3% (n=2356) had a COVID-19 code in HES, and 28.7% (n=950) did not;

### Supplementary table S5 - Cross-coding of COVID-19 in HES and SGSS data within the RECOVERY population

| HES COVID-19 coding and SGSS status in RECOVERY  (March 2020 – November 2021) | | COVID-19 coding in the primary diagnostic position in the HES spell straddling randomisation  (U071 or U072) | | |
| --- | --- | --- | --- | --- |
|  |  | Y | N | Total |
| SGSS status  (Y = positive test; N = negative test or no record) | Y | 32770 (84.2%) | 2844  (7.3%) | 35614  (91.5%) |
|  | N | 2159  (5.5%) | 547  (1.4%) | 3306  (8.5%) |
|  | Total | 34929 (89.7%) | 3391  (10.3%) | 38920  (100%)* |

*all proportions calculated with 38920 (row and column total, corresponding to the total number of participants with available HES data) as denominator

These data show that, in a population admitted to hospital with clinically-proven or suspected COVID-19 (RECOVERY trial participants), using HES as a single source to identify a potential COVID-19 cohort (and restricting to the primary diagnostic position) would capture
~91% of the intended population, with very high agreement between HES COVID-19 coding and the presence of a positive test in SGSS. Nonetheless, this would lead to the inclusion of 8.5% of people with no record of a positive test in SGSS (although the absence of a record in SGSS could be due to either a negative test or the absence of a test result, and SGSS only covers in-hospital testing). Conversely, this approach would lose 10.3% of patients recruited to RECOVERY (and 8.0% of those with a positive in-hospital COVID-19 test). Overall, HES seems to be a reliable way of identifying a population admitted to hospital with COVID-19 (even if not using testing data from SGSS).

1. **U071 vs U072 coding in HES**

COVID-19 can be coded in ICD-10 using either U071 (“COVID-19, virus identified”) or U072 (“COVID-19, virus not identified”); of note, other COVID-19 related codes are available in ICD-10 but these are unrelated to acute diagnosis (see <https://www.who.int/standards/classifications/classification-of-diseases/emergency-use-icd-codes-for-covid-19-disease-outbreak>)

When comparing different COVID-19 coding in HES with SGSS records:

1. The majority of RECOVERY participants with HES records (n=38920) are coded using the U071 code with or without U072 (92.0%, n=35812), with only a minority recorded with the U072 code only (2.9%, n=1125);
2. Of those people with no SGSS record (8.5% of those with HES data available, n= 3306), a significant proportion (54.1%; n=1789) had a record of U071 (with or without U072), 30.6% (n=1012) had a record of U072 only, and 20.1% (n=690) had no COVID-19 coding;
3. Of those people with a U072 record only (n=1251), 239 (19.1%) still had a positive SGSS record.

The value of using U072 is in identifying possible COVID-19 patients versus those with no COVID-19 codes. If this code were employed as intended by its definition, it would be expected that its use, when compared with people with no COVID-19 codes, would be more frequent in SGSS-negative patients (those for whom a test was negative or not performed) versus those SGSS-positive. In the RECOVERY population, the odds of having a U072 code recorded in HES versus no code recorded is 0.3 (239:779) in SGSS-positive patients, and 1.28 (886:690) in SGSS-negative patients (odds-ratio: 4.3); this shows an increased likelihood of U072 use in RECOVERY patients in the absence of a positive test (versus those with a positive test recorded), suggesting a correct use of U072 in cases of suspected COVID-19 with no positive test recorded.

### Supplementary table S6 - Cross-coding of COVID-19 in HES (disaggregated across different ICD-10 codes) and SGSS data within the RECOVERY population

| **HES COVID-19 coding and SGSS status in RECOVERY (March 2020 – November 2021)** | | **COVID-19 coding in any diagnostic position in HES,**  **at any time point**  **(U071 or U072)** | | | | |
| --- | --- | --- | --- | --- | --- | --- |
|  |  | **U071 or U072** | **U071**  **(+/- U072)** | **U072 only** | **None** | **Total** |
| **SGSS status**  **(Y = positive test;**  **N = negative test or no record)** | **Y** | 34466 (88.6%) | 34023 (87.4%) | 239  (0.6%) | 779  (2.0%) | 35614  (91.5%) |
|  | **N** | 2477 (6.4%) | 1789 (4.6%) | 886  (2.3%) | 690  (1.8%) | 3306  (8.5%) |
|  | **Total** | 36943  (94.9%) | 35812 (92.0%) | 1125  (2.9%) | 1469  (3.8%) | 38920 (100%)* |

*all proportions calculated with 38920 (corresponding to the total number of participants with available HES data) as denominator; NB: columns are not mutually exclusive

These observations suggest that U071 is the most widely used code, with only a minority of people coded using U072 only; of these, there is still evidence of a positive COVID-19 test in a significant proportion; moreover, the relative frequency of U072 use in SGSS-positive and SGSS-negative patients points towards correct use by clinical coders. In conclusion, these calculations support the use of U072 alongside U071 in a population admitted to hospital with COVID-19.

Hence, we have defined the national reference population based on the presence of a U071 or U072 code, further restricted to the primary diagnostic position to avoid inclusion of people in whom COVID-19 is not the main reason for care.

## Annex IV - Cross-coding of invasive and non-invasive mechanical ventilation in the RECOVERY case report form versus Hospital Episode Statistics data

Methodology:

- RECOVERY participants randomised in England on or before the 30-11-2021 (analysis period for the current study)
- People with HES data available for whom the most recent episode included in the HES data starts on or after randomisation (i.e. HES data covers the period of randomisation)
- HES records restricted to OPCS codes recorded (procedure date) on or before the day of randomisation (inclusive), and using a lookback period of 15 or 30 days before randomisation in the HES data

Population:

- 47029 distinct participant records in the CRF data
- 46827 after removing withdrawals or duplicates
- 40725 recruited in England
- 39450 randomised within the study period
- 38025 included in the HES data (all of which recruited in England)

### Supplementary table S7 - IMV coding cross tabulation (HES vs CRF) - 15 days before randomisation

| HES data |  | Case report form | | | Total |
| --- | --- | --- | --- | --- | --- |
|  |  | IMV/ECMO | NIV/oxygen | None | All |
| IMV | N | **1023** | 808 | 12 | 1843 |
|  | % col | **39.9** | 2.6 | 0.3 | 4.8 |
| None | N | 1542 | 30062 | 4578 | 36182 |
|  | % col | 60.1 | 97.4 | 99.7 | 95.2 |
| All | N | 2565 | 30870 | 4590 | 38025 |
|  | % col | 100.0 | 100.0 | 100.0 | 100.0 |

### Supplementary table S8 - IMV coding cross tabulation (HES vs CRF) - 30 days before randomisation

| HES data |  | Case report form | | | Total |
| --- | --- | --- | --- | --- | --- |
|  |  | IMV/ECMO | NIV/oxygen | None | All |
| IMV | N | **1125** | 942 | 17 | 2084 |
|  | % col | **43.9** | 3.1 | 0.4 | 5.5 |
| None | N | 1440 | 29928 | 4573 | 35941 |
|  | % col | 56.1 | 96.9 | 99.6 | 94.5 |
| All | N | 2565 | 30870 | 4590 | 38025 |
|  | % col | 100.0 | 100.0 | 100.0 | 100.0 |

### Supplementary table S9 - NIV coding cross tabulation (HES vs CRF) - 15 days before randomisation

| HES data |  | Case report form | | | Total |
| --- | --- | --- | --- | --- | --- |
|  |  | IMV/ECMO | NIV/oxygen | None | All |
| NIV | N | 1305 | 8222 | 129 | 9656 |
|  | % col | 50.9 | 26.6 | 2.8 | 25.4 |
| None | N | 1260 | 22648 | 4461 | 28369 |
|  | % col | 49.1 | 73.4 | 97.2 | 74.6 |
| All | N | 2565 | 30870 | 4590 | 38025 |
|  | % col | 100.0 | 100.0 | 100.0 | 100.0 |

### Supplementary table S10 - NIV coding cross tabulation (HES vs CRF) - 30 days before randomisation

| HES data |  | Case report form | | | Total |
| --- | --- | --- | --- | --- | --- |
|  |  | IMV/ECMO | NIV/oxygen | None | All |
| NIV | N | 1438 | 8948 | 150 | 10536 |
|  | % col | 56.1 | 29.0 | 3.3 | 27.7 |
| None | N | 1127 | 21922 | 4440 | 27489 |
|  | % col | 43.9 | 71.0 | 96.7 | 72.3 |
| All | N | 2565 | 30870 | 4590 | 38025 |
|  | % col | 100.0 | 100.0 | 100.0 | 100.0 |
