# Supplementary material for: Clinical trial results in context: comparison of baseline characteristics and outcomes of 38,510 RECOVERY trial participants versus a reference population of 346,271 people hospitalised with COVID-19 in England
Source: Trials. 2024 Jun 29;25:429. doi: 10.1186/s13063-024-08273-9 (PMC11218071; doi:10.1186/s13063-024-08273-9)
Supplement: Supplementary file 2 — Additional file 2. [file 13063_2024_8273_MOESM2_ESM.docx]

**The RECORD statement**

|  | **Item No.** | **STROBE items** | **Location in manuscript where items are reported** | **Page numbers** | **RECORD items** | **Location in manuscript where items are reported** | **Page numbers** |
| --- | --- | --- | --- | --- | --- | --- | --- |
| **Title and abstract** |  |  |  |  |  |  |  |
|  | 1 | (a) Indicate the study’s design with a commonly used term in the title or the abstract (b) Provide in the abstract an informative and balanced summary of what was done and what was found | Title and abstract | 1,3 | RECORD 1.1: The type of data used should be specified in the title or abstract. When possible, the name of the databases used should be included.  RECORD 1.2: If applicable, the geographic region and timeframe within which the study took place should be reported in the title or abstract.  RECORD 1.3: If linkage between databases was conducted for the study, this should be clearly stated in the title or abstract. | Abstract  Abstract + title  Abstract  Abstract | 1,3  1,3  3  3 |
| **Introduction** |  |  |  |  |  |  |  |
| Background rationale | 2 | Explain the scientific background and rationale for the investigation being reported | Introduction | 5-6 |  |  |  |
| Objectives | 3 | State specific objectives, including any prespecified hypotheses | Introduction | 5-6 |  |  |  |
| **Methods** |  |  |  |  |  |  |  |
| Study Design | 4 | Present key elements of study design early in the paper | Abstract, methods | 3, 8-12 |  |  |  |
| Setting | 5 | Describe the setting, locations, and relevant dates, including periods of recruitment, exposure, follow-up, and data collection | Abstract, methods, supplementary methods, Figure 2 | 3, 8-12, 22  Supplement: 1-3 |  |  |  |
| Participants | 6 | *(a) Cohort study* - Give the eligibility criteria, and the sources and methods of selection of participants. Describe methods of follow-up  *Case-control study* - Give the eligibility criteria, and the sources and methods of case ascertainment and control selection. Give the rationale for the choice of cases and controls  *Cross-sectional study* - Give the eligibility criteria, and the sources and methods of selection of participants  *(b) Cohort study* - For matched studies, give matching criteria and number of exposed and unexposed  *Case-control study* - For matched studies, give matching criteria and the number of controls per case | Methods, supplementary methods (Annex I in the Supplement)  N/A  N/A  N/A  N/A  N/A | 8-11, 21  Supplement: 1-3, 8-11 | RECORD 6.1: The methods of study population selection (such as codes or algorithms used to identify subjects) should be listed in detail. If this is not possible, an explanation should be provided.  RECORD 6.2: Any validation studies of the codes or algorithms used to select the population should be referenced. If validation was conducted for this study and not published elsewhere, detailed methods and results should be provided.  RECORD 6.3: If the study involved linkage of databases, consider use of a flow diagram or other graphical display to demonstrate the data linkage process, including the number of individuals with linked data at each stage. | Methods, Figure 1, supplementary methods (Annex I in the Supplement)  Annex III in the Supplement  Figure 1 | 8-11, 21  Supplement: 1-3, 8-11  Supplement: 53-58  21 |
| Variables | 7 | Clearly define all outcomes, exposures, predictors, potential confounders, and effect modifiers. Give diagnostic criteria, if applicable. | Methods, supplementary methods (annex I) | 10-12; supplement: 3-25 | RECORD 7.1: A complete list of codes and algorithms used to classify exposures, outcomes, confounders, and effect modifiers should be provided. If these cannot be reported, an explanation should be provided. | Methods, supplementary methods (annex I) | Supplement: 3-25 |
| Data sources/ measurement | 8 | For each variable of interest, give sources of data and details of methods of assessment (measurement).  Describe comparability of assessment methods if there is more than one group | Methods, supplementary methods (annex I)  Methods, supplementary methods (annex I) | 8-12; supplement: 1-25  8-12; supplement: 1-25 |  |  |  |
| Bias | 9 | Describe any efforts to address potential sources of bias | Methods, supplementary methods (annex I), annex III | 8-12; supplement: 1-25, 53-58 |  |  |  |
| Study size | 10 | Explain how the study size was arrived at | Results, Figure 1 | 13, 21 |  |  |  |
| Quantitative variables | 11 | Explain how quantitative variables were handled in the analyses. If applicable, describe which groupings were chosen, and why | Methods | 11-12 |  |  |  |
| Statistical methods | 12 | (a) Describe all statistical methods, including those used to control for confounding  (b) Describe any methods used to examine subgroups and interactions  (c) Explain how missing data were addressed  (d) *Cohort study* - If applicable, explain how loss to follow-up was addressed  *Case-control study* - If applicable, explain how matching of cases and controls was addressed  *Cross-sectional study* - If applicable, describe analytical methods taking account of sampling strategy  (e) Describe any sensitivity analyses | Methods  Methods  N/A (only people with follow-up data available were included in the cohort)  N/A (only people with follow-up data available were included in the cohort)  N/A  N/A | 11-12  11-12  8-12, 21  8-12, 21 |  |  |  |
| Data access and cleaning methods |  |  |  |  | RECORD 12.1: Authors should describe the extent to which the investigators had access to the database population used to create the study population.  RECORD 12.2: Authors should provide information on the data cleaning methods used in the study. | Methods, supplementary methods (annex I)  Methods, supplementary methods (annex I) | 8-10, supplement: 1-3  Supplement: 1-25 |
| Linkage |  | .. |  |  | RECORD 12.3: State whether the study included person-level, institutional-level, or other data linkage across two or more databases. The methods of linkage and methods of linkage quality evaluation should be provided. | Methods, supplementary methods (annex I) | 8-10, supplement: 1-3 |
| **Results** |  |  |  |  |  |  |  |
| Participants | 13 | (a) Report the numbers of individuals at each stage of the study (*e.g.*, numbers potentially eligible, examined for eligibility, confirmed eligible, included in the study, completing follow-up, and analysed)  (b) Give reasons for non-participation at each stage.  (c) Consider use of a flow diagram | Results, Figure 1 | 13, 21 | RECORD 13.1: Describe in detail the selection of the persons included in the study (*i.e.,* study population selection) including filtering based on data quality, data availability and linkage. The selection of included persons can be described in the text and/or by means of the study flow diagram. | Methods, Figure 1, supplementary methods (Annex I) | 8-10, 21, supplement: 1-3 |
| Descriptive data | 14 | (a) Give characteristics of study participants (*e.g.*, demographic, clinical, social) and information on exposures and potential confounders  (b) Indicate the number of participants with missing data for each variable of interest  (c) *Cohort study* - summarise follow-up time (*e.g.*, average and total amount) | Results, Table 1, Figure 2, Supplementary tables S1, S2, S4; supplementary figures S1-S6  Table 1, Figure 1, Figure 2, Supplementary figure S3  Methods, Results, Figure 3, Supplementary figures S7-S9 | 13, 14, 22, 23, 24; supplement: 36-49  21,22,24-26; supplement: 46  8-10, 13-14, 23; supplement: 50-52 |  |  |  |
| Outcome data | 15 | *Cohort study* - Report numbers of outcome events or summary measures over time  *Case-control study* - Report numbers in each exposure category, or summary measures of exposure  *Cross-sectional study* - Report numbers of outcome events or summary measures | Results, Figure 3, Supplementary tables S4-S5, Supplementary figure S7  N/A  N/A | 13-14, 23; supplement: 50-52 |  |  |  |
| Main results | 16 | (a) Give unadjusted estimates and, if applicable, confounder-adjusted estimates and their precision (e.g., 95% confidence interval). Make clear which confounders were adjusted for and why they were included  (b) Report category boundaries when continuous variables were categorized  (c) If relevant, consider translating estimates of relative risk into absolute risk for a meaningful time period | Methods, Results, Figure 3, Supplementary tables S3-S4, Supplementary figures S7-S9  Methods, Table 1  N/A | 11-12,13-14, 23; supplement: 42,43, 50-52  11-12, 24-26 |  |  |  |
| Other analyses | 17 | Report other analyses done—e.g., analyses of subgroups and interactions, and sensitivity analyses | Methods, Results, Figure 3, Supplementary tables S1-S4, Supplementary figures S1-S9 | 11-12, 13-14; supplement: 36-52 |  |  |  |
| **Discussion** |  |  |  |  |  |  |  |
| Key results | 18 | Summarise key results with reference to study objectives | Discussion | 15 |  |  |  |
| Limitations | 19 | Discuss limitations of the study, taking into account sources of potential bias or imprecision. Discuss both direction and magnitude of any potential bias | Discussion | 16-17 | RECORD 19.1: Discuss the implications of using data that were not created or collected to answer the specific research question(s). Include discussion of misclassification bias, unmeasured confounding, missing data, and changing eligibility over time, as they pertain to the study being reported. | 16-17 |  |
| Interpretation | 20 | Give a cautious overall interpretation of results considering objectives, limitations, multiplicity of analyses, results from similar studies, and other relevant evidence | Discussion, conclusion | 16-17 |  |  |  |
| Generalisability | 21 | Discuss the generalisability (external validity) of the study results | Discussion, conclusion | 16-17 |  |  |  |
| **Other Information** |  |  |  |  |  |  |  |
| Funding | 22 | Give the source of funding and the role of the funders for the present study and, if applicable, for the original study on which the present article is based | Declarations | 19 |  |  |  |
| Accessibility of protocol, raw data, and programming code |  | .. | Methods, declarations supplementary methods (annex I) | 8-10,18, supplement: 26-34 | RECORD 22.1: Authors should provide information on how to access any supplemental information such as the study protocol, raw data, or programming code. | Methods, data availability statement | 8-10,18, supplement: 26-34 |

*Reference: Benchimol EI, Smeeth L, Guttmann A, Harron K, Moher D, Petersen I, Sørensen HT, von Elm E, Langan SM, the RECORD Working Committee. The REporting of studies Conducted using Observational Routinely-collected health Data (RECORD) Statement. *PLoS Medicine* 2015; in press.

*Checklist is protected under Creative Commons Attribution ([CC BY](http://creativecommons.org/licenses/by/4.0/)) license.
